# Targeting Intracranial Tumours with a Combination of RNA and Chemotherapy

**DOI:** 10.3390/pharmaceutics16060829

**Published:** 2024-06-18

**Authors:** Abdulhamid S. Fatani, Andreas G. Schätzlein, Ijeoma F. Uchegbu

**Affiliations:** 1 UCL School of Pharmacy, 29-39 Brunswick Square, London WC1N 1AX, UK; dr.fatani1@hotmail.com (A.S.F.); a.schatzlein@ucl.ac.uk (A.G.S.); 2 Nanomerics Ltd., Block Y, Northwick Park and St Mark’s Hospital, Watford Road, Harrow HA1 3UJ, UK

**Keywords:** siRNA anti-*ITCH*, 6-O-glycolchitosan, brain, intracranial tumours, intranasal, brain delivery, gene therapy

## Abstract

Glioblastoma multiforme (GBM) is a fast-growing and aggressive brain tumour, which remains largely resistant to treatment; the prognosis for patients is poor, with a median survival time of about 12–18 months, post diagnosis. In an effort to bring more efficacious treatments to patients, we targeted the down regulation of ITCH, an E3 ligase that is overexpressed in a variety of cancers, and which inhibits P73, a tumour suppressor gene. 6-O-glycolchitosan (GC) was used to deliver siRNA ITCH (GC60-siRNA-ITCH) and gemcitabine via the nose to brain route in CD-1 nude mice which had previously been implanted intracranially with U87-MG-luc2 cells. Prior to this in vivo study, an in vitro study established the synergistic effect of siRNA-ITCH in combination with a chemotherapy drug—gemcitabine. A downregulation of ITCH, an upregulation of p73 and enhanced apoptosis were observed in vitro in U87-MG cells, using qPCR, Western blot analysis, confocal laser scanning microscopy, flow cytometry and cytotoxicity assays. When GC60-siRNA-ITCH was combined with gemcitabine, there was a resultant decrease in cell proliferation in vitro. In CD1 mice, the administration of siRNA-ITCH (7 doses of 0.081 mg/kg) alone did not significantly affect animal survival (increasing mean survival from 29 to 33 days when compared to untreated animals), whereas intranasal gemcitabine had a significant effect on survival (increasing survival from 29 to 45 days when compared to untreated animals, *p* < 0.01). The most significant effect was seen with combination therapy (GC60-siRNA-ITCH plus gemcitabine), where survival increased by 89%, increasing from 29 to 54 days (*p* < 0.01). Our data demonstrate that siRNA chemosensitises brain tumours to gemcitabine and that the nose-to-brain delivery route may be a viable route for the treatment of intracranial tumours.

## 1. Introduction

The prognosis for glioblastoma multiforme (GBM) patients is very poor, with median survival, post treatment, not exceeding 15 months [1]. Surgery and radiotherapy followed by maintenance chemotherapy with temozolomide is the standard of care [1]. GBM is characterised by uncontrolled cellular proliferation, diffuse infiltration and significant angiogenesis [2]. For some GBM tumours, there is no acceptable treatment [3,4]. Chemotherapy, when indicated, is very challenging, due to the heterogeneous and infiltrating nature of tumours, and due to the fact that the blood–brain barrier prevents chemotherapeutic agents from accumulating at the tumour site [2,5,6]. Therefore, new methods to enable therapeutics to access brain tumours are required. We have demonstrated that plasmids [7,8] and small interfering ribonucleic acids [9], formulated as polyplexes, may be delivered to the brain tissue using the intranasal route of delivery and that genes are actually targeted to the cerebral cortex on intranasal delivery [7,8]. These nucleic acids travel through the olfactory bulb and distribute to the larger sections of the brain by processes that are not particularly well understood, but we speculate that the brain distribution processes may involve perivascular mechanisms, as proposed by others [10]. With this knowledge, we hypothesised that the nose-to-brain route [11] may be used to localise chemotherapy at the tumour site, and demonstrating the effects of such drug targeting is the aim of our work. We opted to use a polymeric delivery system that is more biocompatible than the commercial lipid formulation, Lipofectamine [8]. 

ITCH and other members of the Nedd4 class of proteins share similar functional responsibilities for ubiquitination and degradation of multiple tumour suppressor proteins, including p73 and p63 [12,13]. The p73 and p63 tumour suppressors belong to the same family as p53 and share the same functions as p53; namely, inducing apoptosis following cellular stress and DNA damage, and are considered targets for ITCH, thus suggesting a critical role for ITCH in the regulation of apoptosis [12,13,14,15]. The human ITCH gene is highly overexpressed in anaplastic thyroid carcinomas (ATCs) at chromosome 20q11.22 [16]. The overexpression of the ITCH gene is correlated with proliferation of ATC cells, and ITCH gene silencing using small interfering RNA (siRNA) decreased the proliferation and induced apoptosis of ATC cells [16]. We have previously shown that the down regulation of the ITCH gene increased the chemosensitivity to gemcitabine in a pancreatic cancer xenograft (MIA PaCa-2) mouse flank tumour, with a resultant increased tumour response to gemcitabine [17]. We hypothesise that targeting the ITCH gene could become a viable therapeutic strategy for treating GBM, and for improving chemo-sensitivity generally in chemotherapy. 

Gemcitabine is a nucleoside analogue that possesses broad-spectrum anti-tumour activity and is approved for use in pancreatic, breast, ovarian and lung cancer [18,19,20]. Intracellular conversion (phosphorylation) of gemcitabine to the active diphosphate (dFdCDP) and triphosphate (dFdCTP) nucleosides by the deoxycytidine kinase (DCK) enzyme leads to the competitive inhibition of DNA polymerase and the inhibition of DNA synthesis [21,22]. In addition, dFdCTP, the gemcitabine metabolite, may be incorporated into the DNA helix as a false nucleoside, preventing the replication of DNA and inducing the destruction of DNA during the synthesis phase (S phase) of the cell cycle [23]. Several types of cancers develop either complete or partial resistance to gemcitabine, and this is one of the biggest challenges when using gemcitabine treatments [19]. Gemcitabine is hydrophilic and this prevents it from crossing cellular membranes via passive diffusion; hence, it depends on an active internalisation mechanism through nucleoside transporters [24]. As such, resistance to gemcitabine may also develop from the altered expression of nucleoside transporters in cell membranes [25]. Resistance may also develop due to changes in the enzymes responsible for DNA repair and polymerisation [25]. Intracellularly, insufficient DCK activity causes resistance since the initial step of gemcitabine phosphorylation is critical to triggering the pharmacological activity of gemcitabine in the entire phosphorylation cascade [26]. A further source of resistance to gemcitabine lies in the deamination of dFdCMP and gemcitabine, catalysed by the overexpression of intracellular deoxycytidylate deaminase (DCTD) and cytidine deaminase (CDA), respectively [20]. In general, alterations in the nucleoside transporter, a lack of DCK activity and CDA overexpression are considered the major causes of the resistance against gemcitabine [19].

Gemcitabine also has a short in vivo half-life. On intravenous injection, gemcitabine is promptly deaminated to 2,2-difluorodeoxyuridine (dFdU) by CDA, an enzyme which is found in the blood and liver [27]. Deamination is followed by rapid renal clearance [27]. Due to the low bioavailability and short half-life of gemcitabine in the plasma (approximately 10 min), repeated administration is needed; repeated administration is associated with multiple side effects such as cutaneous toxicity, oedema, thrombocytopenia, myelosuppression, nephrotoxicity and hepatotoxicity, and it does not result in sufficient therapeutic effectiveness [28,29,30]. In addition, the limited penetration of gemcitabine into some solid tumours leads to increased resistance and reduced efficacy [30,31,32]. The intranasal delivery of gemcitabine is a novel delivery approach, designed to overcome the peripheral side effects outlined above. Enhancing the activity of gemcitabine via pro-apoptosis mechanisms (such as down regulation of the ITCH gene and concomitant up regulation of the pro-apoptotic gene, p73) has been shown to improve the tumouricidal activity of gemcitabine in mouse flank tumours [17], a process known as chemosensitisation. Chemosensitisation is defined as using one drug to enhance the activity of another selectively in tumour cells, while limiting any undesired side effects and toxicity in normal cells [33]. In this paper, we present in vitro and nose-to-brain in vivo data on the inhibition of ITCH expression using siRNA-ITCH and the resultant effects on tumour cell proliferation when combined with gemcitabine, and we explore the mechanisms underpinning the observed effects. 

## 2. Materials and Methods

### 2.1. Materials

A U87-MG cell line was purchased from American Type Culture Collection (ATCC, Manassas, VA, USA). The number of passages was between 19 and 25. An anti-ITCH siRNA (sense strand: 5′ GCU-GUU-GUU-UGC-CAU-AGA-A55 3′; antisense strand: 5′ UUC-UAU-GGC-AAA-CAA-CAG-C 3′) and scrambled siRNA were obtained from Euphoria Biotech, UK (Dresden, Germany). The annexin V Alexa Fluor^TM^ 488 conjugate/propidium iodine (PI) double-staining kit was obtained from Thermo Fisher Scientific, UK (Oxford, UK).

Eagle’s Minimal Essential Medium Eagle (EMEM), Foetal Bovine Serum (FBS), Penicillin/Streptomycin, Trypsin (0.25 *w*/*v* with 0.53 mM EDTA), Trypan Blue and Protease & Phosphatase Inhibitor Cocktails were supplied by Sigma Aldrich Chemical Company (Dorset, UK). Gibco^TM^ Sodium Pyruvate, Gibco^TM^ GlutaMAX^TM^ and Piecer Rapid Gold BCA Protein Assay Kit were supplied by Thermo Fisher, (Loughborough, UK). Dulbecco’s Phosphate Buffered Saline (DPBS) was supplied by Gibco (Dorset, UK). The U87 cells (ATCC, HTB—14^TM^) and Bioluminescent U87-MG-Luc2 cells were supplied by ATCC (ATCC, Teddington, UK) and (ATCC, Manassas, VA, USA), respectively. The PCR reagents, including RNeasy mini kit, QIA shredder spin column, Super Script^TM^ III First-Strand Synthesis System, SYBR^TM^ Green Master Mix in an AriaMx Realtime PCR System, PCR Primers (ITCH, P73 and Actin), RNA Lysis Buffer, RNA Wash Buffer and RNA Wash Buffer with Ethanol were supplied by (Qiagen, Manchester, UK). A diluted binding buffer, Propidium Iodide (PI) and Annexin V were supplied by Thermo Fisher, Invitrogen (Loughborough, UK). The tank buffer (25 mM Tris, 192 mM Glycine, 0.1% SDS, pH 8.3) and the transfer buffer (25 mM Tris, 192 mM glycine, 20% (vol/vol) methanol, pH 8.3) were supplied by (Bio-Rad, Watford, UK). D-luciferin (VivoGlo^TM^ Luciferin in vivo grade) was supplied by Promega (Southampton, UK). Female CD-1 nude mice (10–12 weeks old, 27–30 g) were obtained from Charles River, (Oxford, UK).

### 2.2. Methods

#### 2.2.1. Polyplex Preparation

Polyplexes were prepared by adding siRNA-ITCH (0.020 mg/mL in 20 mM sodium triphosphate, pH = 6.8, 1 mL) to GC60 (2 mg/mL in 20 mM sodium triphosphate buffer, pH = 6.8, 1 mL) and mixing with a pipette for 10 s. GC60-siRNA-ITCH polyplexes were always prepared at a GC60, siRNA ratio of 100:1. GC60-siRNA-ITCH polyplexes were incubated for 24 h, at 4 °C prior to use. Polyplexes were imaged using transmission electron microscopy, using methods described previously [7].

#### 2.2.2. Gene Silencing In Vitro

##### Transfection Experiments

U87-MG cells were cultured with Eagle’s Minimal Essential Medium Eagle (EMEM) supplemented with sodium pyruvate solution (1% *v*/*v*), GlutaMAX (1% *v*/*v*) foetal bovine serum (FBS, 10% *v*/*v*) and a penicillin (10,000 U/mL)—streptomycin (100 μg/mL) solution; the cells were incubated at 37 °C in a humidified atmosphere containing 5% CO_2_. U87-MG cells were seeded at a density of 5 × 10^5^ cells/well in a 6-well plate and incubated for 48 h. The cells were then separately treated with 400 nM of siRNA (5 μg/well siRNA) complexed with either GC60 (GC60, siRNA, ratio = 100:1 g/g) or Lipofectamine 2000 complexes (Lipofectamine 2000, siRNA, ratio = 2:1 g/g) for 48 h or 72 h and following the manufacturer’s transfection protocol [34]. Cells were then analysed by flow cytometry, confocal laser scanning microscopy, qPCR and/or Western blot analysis. 

##### Flow Cytometry

In this assay, apoptotic cells were detected using the Annexin V Alexa Fluor^TM^ 488 conjugate (Annexin V)/propidium iodine (PI) double-staining kit. Briefly, U87-MG cells (500,000 cells per well) were treated with different siRNA formulations for 48 h (10 μg per well) as outlined above, then detached by trypsin (0.25% *w*/*v*, 4 mL) containing ethylene diamine tetra acetic acid (EDTA, 2.21 mM), pelleted using a Centrifuge 5430 (Eppendorf, Hamburg, Germany) at 400 rpm for 4 min at 20 °C, and washed three times in Dulbecco’s Phosphate Buffered Saline (DPBS—without Ca^2+^ and Mg^2+^). Cells were then resuspended in 100 μL of a diluted binding buffer supplied by the manufacturer. After adding PI (50 μg/mL, 5 μL) and Annexin V (20 μg/mL, 5 μL), the labelled cells were incubated at room temperature for 10 min in the dark. 

In addition, a second set of U87-MG cells (500,000 cells/well in a 6-well plate) were treated with gemcitabine (33 mg/mL, 0.06 mL) and formulations of siRNA-ITCH (0.1 mg/mL, 0.016 mL) or scrambled siRNA (0.1 mg/mL, 0.016 mL) for 48 h, then detached using trypsin (0.25% *w*/*v*, 4 mL) containing EDTA (2.21 mM), and pelleted by centrifugation at 400 rpm for 4 min at 20 °C. The cells were then washed three times in DPBS (without Ca^2+^ and Mg^2+^) and resuspended in 100 μL of a diluted binding buffer. After adding PI (50 μg/mL, 5 μL) and Annexin V (20 μg/mL, 5 μL), the cells were incubated at room temperature for 10 min in the dark. The level of apoptosis was analysed using a fluorescence-activated single cell sorting (FACS) flow cytometer equipped with a CyAn^TM^ Advanced Digital Processing (ADP) analyser (Beckman Coulter, Brea, CA, USA). The data were analysed using Summit software version 6 (Summit Company, Maumelle, AR, USA). 

##### Real-Time Polymerase Chain Reaction (RT-PCR) Assays 

Real-time quantitative reverse transcription-polymerase chain reaction (RT-qPCR) was used to quantify mRNA [ITCH, P73 and GAPDH (the housekeeping gene)] in U87-MG cells after treatment with siRNA-ITCH. U87-MG cells were seeded at a density of 5 × 10^5^ cells/well in a six-well plate and allowed to grow for 48 h. The cells were then separately treated with 400 nmol of siRNA (5 μg/well siRNA) either as the G60 polyplex (GC60, siRNA = 100:1 g/g) or a Lipofectamine 2000 lipoplex formulation (Lipofectamine 2000, siRNA = 2:1 g/g). Alternatively, one group of cells was treated with GC60-scrambled siRNA polyplexes prepared in exactly the same way as the GC60-siRNA-ITCH polyplexes. Cells were treated for 48 h prior to being lysed and extracted using the RNeasy mini kit according to the manufacturer’s instructions (Qiagen, Manchester, UK). 

U87-MG cells were lysed with 350 μL of RNA Lysis Buffer (a lysing buffer solution supplied by the manufacturer containing 1% beta-mercaptoethanol) and then pipetted up and down to ensure thorough mixing with the transfected cells. The resulting cell lysates were passed through a QIAshredder spin column fixed in a 2 mL collection tube (Qiagen, Manchester, UK) and then centrifuged at 14,000 rpm for 2 min at 20 °C (Mikro 200 Microliter Tube Package 24 Micro-Centrifuge, Hettich company, Kirchlengern, Germany). The QIAshredder spin column (Qiagen, Manchester, UK) was used as an optimising step for homogenising cell lysates in order to harvest the cellular RNA. Ethanol (70% *v*/*v*, 1 mL) was then pipetted into the 2 mL collection tube comprising the cell lysates. Following this, an aliquot (700 μL) of the cell lysate—ethanol mixture was pipetted into a RNeasy spin column for centrifugation at 14,000 rpm for 30 s at 20 °C. The precipitate was removed, and 700 μL of RNA Wash Buffer (a washing buffer), was added to the RNeasy spin column before centrifugation at 14,000 rpm for 30 s at 20 °C. The following steps were applied to achieve two washing steps: an aliquot of the second RNA Wash Buffer with ethanol buffer (500 μL) was added to the RNeasy spin column and this was followed by two centrifugation rounds at 14,000 rpm at 20 °C. The first centrifugation was carried out for 30 s and a second aliquot of RNA Wash Buffer with ethanol (500 μL) added and the second centrifugation was carried out for 2 min. Following these centrifugation steps, RNase-free water (40 μL) was added to the RNeasy spin column to elute the RNA by centrifugation at 14,000 rpm for 1 min at 20 °C. A spectrophotometer (NanoDrop 2022, Thermo Fisher Scientific, Loughborough, UK) was used to quantify the total collected RNA at a wavelength of 260 nm. The extracted RNA was transcribed reversely into cDNA using a SuperScript^TM^ III First-Strand Synthesis System, following the manufacturer’s instructions. The RT-qPCR reaction was achieved using an Eppendorf Mastercycler^®^ machine X50p Aluminium Block for 96-well plates (Hamburg, Germany) with 50 ng of cDNA template and 150 nM of three primers using Power SYBR^TM^ Green Master Mix in an AriaMx Realtime PCR System (Qiagen, Manchester, UK).

GAPDH-forward (TTGCCCTCAACGACCACTTT) and reverse (TGGTCCAGGGGTCTTACTCC).

The cycling involved heating at 95 °C for 15 min to activate polymerase, followed by 40 cycles of denaturation for 15 s at 95 °C and annealing for 60 s at 60 °C (5). The melting curve was analysed between 65 °C and 95 °C. The resulting data originated from three independent experiments analysed in Eppendorf’s Cycle Manager X50 software (Hamburg, Germany). The relative level of mRNA expression was then normalised versus the GAPDH (the housekeeping gene).

##### Western Blotting

U87-MG cells were seeded at a density of 5 × 10^5^ in a 6-well plate. After treatment with siRNA formulations, cells were washed with cold phosphate-buffered saline (PBS, pH = 7.4, 2 mL). The PBS was discarded, and ice-cold Radioimmunoprecipitation buffer (RIPA buffer, 200 µL) supplied by the manufacturer and containing a cocktail of phosphatase and protease inhibitors, was added to each well. The plates were incubated on ice for 20 min, with shaking every 5 min. A plastic cell scraper was then used to transfer the cells into a 1.5 mL sterile tube that had been kept on ice for 10 min. The cell lysates were directly centrifuged (12,000 rpm for 6 min at 4 °C). The supernatants were separated and assayed for protein down regulation by Western blot analyses. 

Protein content from the cell lysates was quantified using a Bicinchoninic acid assay (BCA), and a bovine serum albumin (BSA) standard curve used (prepared between 0.056–2 mg/mL). The BCA method for protein quantification was carried out by taking aliquots (2 µL) of BSA standards or suitably diluted protein samples and adding these to 200 µL water in Eppendorf tubes. The BCA reagent (200 µL) was then added to each sample and mixed well. Once mixed, samples were incubated for 5–10 min at room temperature. Aliquots (200 µL) of each sample were then transferred to 96-well plates and absorbance was measured at a wavelength of 595 nm in a plate reader (PHERAstar, BMG LABTECH, Ortenberg, Germany). The unknown protein concentrations were calculated using the linear equation derived from the calibration curve.

Gels were run at 100 V in a tank buffer (sodium dodecyl sulphate (0.1%), Glycine (192 mM) in Tris buffer (25 mM), pH = 8.3) for 1 h or at least until the bromophenol blue dye front reached the bottom of the gel. Tris buffer consists of NaCl (150 mM) and Tris (20 mM) made to the required pH with NaOH (0.1 M) or HCL (0.1 M). Once electrophoresis was completed, the gel was carefully removed from the cassette and transferred onto a nitrocellulose membrane (0.45 μm; Bio-Rad). A typical sponge–paper–membrane–gel–paper–sponge transfer sandwich was assembled to transfer proteins. The sponges, blotting papers and membrane were soaked in the transfer buffer for at least 10 min prior to the assembly of the transfer sandwich. Blotting took place in the voltaged tank filled with the cold transfer buffer (glycine (192 mM), Tris (25 mM) all in 20% *v*/*v* methanol, pH = 8.3) for 75 min at 100 V.

Membranes were then incubated with blocking buffer containing Tris-buffered saline with Tween 20 (0.1% *w*/*v*), pH = 7.4, (TBST) and containing 5% non-fat milk, for 1 h at room temperature. After that, the membranes were incubated separately with the primary mouse monoclonal antibodies against ITCH (at 1:1000 dilution) and actin (at 1:1000 dilution) at 4 °C overnight. All dilutions were carried out in TBST, pH = 7.4. The membranes were subsequently washed with TBST (pH = 7.4) three times and then, incubated with mouse anti-human IgG secondary antibodies (at 1:1000 dilution) conjugated with horseradish peroxidase (HRP) at room temperature for 1 h. The membranes were then washed three times with TBST buffer (pH = 7.4). A SuperSignal^TM^ West Femto Maximum Sensitivity chemiluminescent substrate kit (Thermo Fisher Scientific, Oxford, UK) was used to catalyse HRP with luminol to generate luminescence. Then the membrane was imaged by a ChemiDoc^TM^ MP system (Bio-Rad, Watford, UK) and analysed using Image Lab software version 6.1 (Bio-Rad). 

##### Confocal Microscopy

Cell samples (2.5 × 10^5^ cells per mL, 0.1 mL) were incubated with 4′-,6-diamidino-2-phenylindole (DAPI, 1 mg/mL, 4 drops in 2 mL) for 30 min prior to the experiments to allow visualization of the nuclei. The supernatant was removed after centrifuging the cells at 400 rpm for 4 min at room temperature. The cell pellet was gently resuspended in 0.5 mL of the manufacturer’s Binding Buffer. To the cell suspension was added annexin V (5 μL) and PI (5 μL) and the cells were incubated for 10 min at room temperature in the dark, with imaging following immediately thereafter using an Axiovert S100 inverted microscope equipped with an oil immersion 40X objective (Carl Zeiss GmbH, Oberkochen, Germany).

##### Cytotoxicity Studies

The U87-MG cells were seeded at a density of 5000 cells/well in a 96-well plate and left to recover for 48 h, before being incubated with a mixture of siRNA-ITCH (8 μg/mL) and various concentrations of gemcitabine (serially diluted from a stock solution of 50 mg/mL gemcitabine) for 48 h. These treated cells were compared to cells that were also incubated with similar concentrations of gemcitabine and the control scrambled siRNA (8 μg/mL) for 48 h. Then, the treatments were discarded, and the cells were replenished with 200 μL of serum-rich medium (EMEM) followed by 48 h or 72 h of recovery. Subsequently, 200 μL of MTT-containing media (5 mg/mL) was added to each of the 96 wells and incubated at 37 °C for 2 h. The media containing MTT solution was then discarded and replaced with dimethyl sulfoxide (DMSO) to lyse the cells, and the 96-well plate was shaken at room temperature for 15 min. The absorbance was measured at 570 nm using an ELx808 absorbance microplate reader (BioTek Instruments, Potton, UK). The cell viability following treatment with the test materials was then calculated with respect to the viability shown by control cells receiving no treatment. 

#### 2.2.3. Animal Studies

##### Tumour Xenograft Studies

Bioluminescent U87-MG-Luc2 cells (ATCC, VA, USA) were incubated at a temperature of 37 °C and in 5% CO_2_ atmosphere in T75 flasks with vented caps in EMEM supplemented with sodium pyruvate solution (1% *v*/*v*), GlutaMAX (1% *v*/*v*), foetal bovine serum (FBS, 10% *v*/*v*) and a penicillin (100 units/mL)—streptomycin (100 μg/mL) solution. The cell passage number was 25. For in vivo tumour implantation, U87-MG-Luc2 cells at 80–85% confluence were detached using a trypsin—EDTA solution (trypsin = 0.25% *w*/*v*, EDTA = 0.53 mM). Cells were then centrifuged (2000 rpm) at 4 °C for 5 min). After discarding the supernatant, the pellet was resuspended in PBS (PH = 7.4) to a final concentration of 50,000 cells per 2 μL in a 0.5 mL Eppendorf tube and placed on an ice bucket until they were implanted. Female CD-1 nude mice weighing between 25 g and 30 g were housed in ventilated cages in groups of 4 mice in each cage and acclimatised for 7 days in a pathogen-free and sterile laboratory environment in the Biological Services Unit (BSU) at University College London.

The study was approved by the local ethics committee and conducted under a UK Home Office Licence, as specified in the Animals (Scientific Procedures) Act 1986 UK. CD-1 mice were anaesthetised using a chamber supplied with 5% inhaled isoflurane and oxygen and then transferred and fixed in a Digital Stereotaxic Apparatus (Harvard Apparatus Company, Cambridge, UK) with a mouse adaptor and mounted on the heat mat. The inhaled isoflurane was then reduced to 2% to maintain the vital signs and the breathing rate was monitored (~60 breaths per minute). The eyes were covered with an eye tears lubricant (Chemist Direct, UK) to prevent desiccation. After cleaning the surgical site on the head with chlorhexidine and subcutaneously injecting Marcaine (bupivacaine 5 mg/mL, 100 μL) under the skin of the head and administering an intraperitoneal injection of Rimadyl (Carprofen 1.5 mg/mL, 100 μL), an incision was made between the eyes towards the back of the cranium.

Under a surgical microscope (Avante Company, London, UK), the bregma (the intersection of the sagittal and coronal sutures) was determined. A hole was drilled in the skull with the coordinates +0.5 mm anterior and 2.25 mm lateral (right) to the bregma (determined by a Harvard Apparatus^TM^ digital stereotaxic control panel), using a sterile surgical micro-drill (Harvard Apparatus, Holliston, MA, USA). A sterile 5 μL Hamilton syringe loaded with 2 μL of bioluminescent U87-MG-Luc2 (50,000 cells) cells, suspended in medium (DPBS—without Ca^2+^ and Mg^2+^) was smoothly lowered through this hole to a depth of 3 mm below the skull surface. Subsequently, the bioluminescent U87-MG-Luc2 cells were injected at a speed of 0.3 μL/min, after which the Hamilton syringe was left at 3 mm depth for 5 min. After gently withdrawing the Hamilton syringe, the incision was sutured using a 5–0 Ethicon Prolene non-absorbable sterile surgical suture (Ethicon, NJ, USA). Then, DPBS (without Ca^2+^ and Mg^2+^, 0.02 mL) was administered by intraperitoneal injection to reduce the effect of the anaesthesia after the surgery. The mice were observed for 15 to 20 min in the observation chamber supplied with oxygen. 

The following day, the mice were weighed and assessed for any signs of pain or distress and had access to the mashed food and sterile water supplied with Rimadyl (Carprofen 1.5 mg/mL) for two additional days. 

On Day 4 after tumour implantation, CD-1 nude mice were randomly allocated to four groups (n = 4 per group) and administered various intranasal formulations. The first group served as the study control and intranasally received normal saline (0.03 mL). The second group was intranasally dosed with GC60-siRNA-ITCH (GC60, siRNA-ITCH 100:1) polyplexes (siRNA = 0.081 mg/kg) alone. The third group was intranasally treated with gemcitabine (33.3 mg/kg) and GC60 (GC60, gemcitabine = 2:1 g/g), scrambled siRNA polyplexes (prepared in exactly the same way as the siRNA-ITCH polyplexes), while the fourth group was intranasally administered with a combination of gemcitabine (33.3 mg/kg) and GC60-siRNA-ITCH (GC60, siRNA-ITCH 100:1) polyplexes (siRNA = 0.081 mg/kg). Mice were dosed daily with siRNA formulations from Day 4 to Day 10 inclusive and were dosed with gemcitabine on Days 4, 7 and 10. When mice were dosed with both siRNA polyplexes and gemcitabine on the same day, there was a 6 h gap between the dose of gemcitabine followed by the dose of siRNA polyplexes. Mice were monitored for any signs of adverse events or severe distress using a clinical score sheet (Figure S1—Supplementary Information). Mice presenting with a weight loss ≥ 15% of their initial body weights or a higher score in the distress sheets (>20), whichever came earlier, were euthanised either by neck dislocation or by using a CO_2_ chamber. The brains were then collected and fixed in formalin for 24 h prior to histopathological sectioning and staining.

The in vivo luminescence imaging was performed with an IVIS system (IVIS^®^-Spectrum systems Xeno-gen-Caliper Life Sciences, Hopkinton, MA, USA) on day 25 post- intracranial implantation of U87-MG-Luc2 the cells. Before imaging, D-luciferin (150 mg/mL, 0.1 mL) was intraperitoneally injected into mice. Then, luciferase expression in Luciferase-labelled U87-MG cells was imaged with the IVIS system 15 min after the D-luciferin (150 mg/kg) injection. The substrate was used as a control to establish a baseline for image analysis with the diverse treatment groups. The machine was connected to a computer with Living Image^®^ 3.0 software, Version 6.1 (Waltham, MA, USA). Image exposure was for 30 s and images were analysed using the Living Image^®^ software and compared based on the same exposure time. A region of interest (ROI) was drawn on the region of the expression signal, and the average counts were measured. The mice were then killed and their brains, livers and lungs were collected. Tissues were immersed immediately in the formalin for 24 h, then washed and transferred to PBS and stored in 4 °C, until histology analysis could be carried out on the tissues. 

##### Histology

Tissues were cut either sagittal or coronal sections, then manually fixed in paraffin wax and left for 1 h to cool in the histology cassette (Fisher Scientific Company, Loughborough, UK). Paraffin-wax-embedded specimens of different mice brain sections were de-paraffinised by immersing slides in xylene twice for 5 min each, absolute ethanol twice for 5 min each, ethanol (95%) for 5 min, ethanol (70%) for 5 min and then, finally, ethanol (50%) for 5 min. After that, the slides were rehydrated by immersing them in distilled water. The slides were then ready for staining. For the haematoxylin and eosin (H&E) procedure, the protocol was performed according to Cardiff’s protocol (28). The slides were stained with haematoxylin for 3 min and eosin Y for 2 min. The tissue slides were then digitally scanned using a scanner (Nano Zoomer S360, Hamamatsu, Japan).

#### 2.2.4. Statistical Analysis 

All data sets were analysed using one-way analysis of variance (ANOVA) for multiple comparisons, with the Tukey multiple comparisons test or two sample *t*-test for a two-sample comparison (Origin 2022 software, Origin Laboratory Corp, Northampton, MA, USA). All data are presented as mean ± standard deviation (SD). A *p*-value below 0.05 was considered statistically significant. * *p* < 0.05, ** *p* < 0.01, *** *p* < 0.001.

## 3. Results

### 3.1. Polyplexes

**Figure 1:**
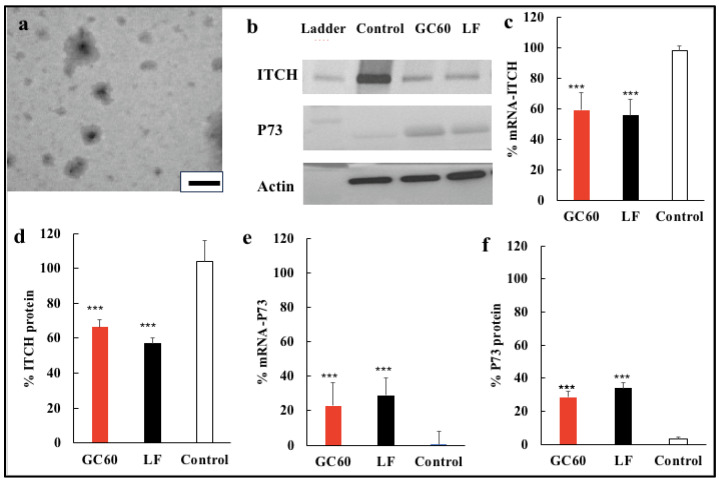
Electron micrograph and in vitro down regulation of ITCH. (**a**) Transmission electron micrograph (TEM) of GC60-siRNA-ITCH (G60, siRNA-ITCH ratio = 100:1 g/g) in phosphate buffer, pH = 6.8 (0.02 mg/mL siRNA), scale bar = 100 nm. (**b**) Western blot gel showing the downregulation of ITCH and up regulation of p73, on application of siRNA-ITCH formulations, 3 days after transfection in U87MG cells: GC60 (GC60, siRNA-ITCH = 100:1 g/g), LF (Lipofectamine 2000, siRNA-ITCH = 2:1 g/g) and Control (G60, siRNA-scrambled = 100:1 g/g) showing the downregulation of ITCH with siRNA-ITCH and the upregulation of p73. Scrambled siRNA formulations show no upregulation of p73. (**c**) ITCH mRNA levels after the application of siRNA-ITCH (GC60 and LF) and Control (scrambled siRNA) formulations, as described above, applied to U87MG cells, and quantified by RT-qPCR. (**d**) ITCH protein expression in Western blot gels after the application of siRNA-ITCH (GC60 and LF) and Control (scrambled siRNA) formulations, as described above, applied to U87MG cells, and quantified using Image J software version 6.1. (**e**) P73 mRNA levels after the application of siRNA-ITCH (GC60 and LF) and Control (scrambled siRNA) formulations, as described above, applied to U87MG cells, and quantified by RT-qPCR, normalised to GAPDH cell mRNA. (**f**). p73 protein expression in Western blot gels after the application of siRNA-ITCH (GC60 and LF) and Control (scrambled siRNA) formulations, as described above, applied to U87MG cells, and quantified using Image J software, normalised to the control. Scrambled siRNA formulations show no downregulation of ITCH and no upregulation of p73 in panels (**e**,**f**). Data are presented as mean ± s.d. (n = 3) from three independent experiments. One-way ANOVA with Tukey’s multiple comparison test was performed and *** = *p* < 0.001 when compared to control treated cells.

GC60 polyplexes (Figure 1a), were imaged after incubation for 24 h at 4 °C and were observed to be spherical, 468 ± 66 nm in diameter and with a zeta potential of +25 ± 2.4 mV at a pH of 6.8 in phosphate buffer, as previously reported [7,35]. 

### 3.2. Gene Silencing In Vitro in U87MG Cells and up Regulation of p73

In order to determine the ability of siRNA-ITCH to specifically downregulate ITCH and then, in turn, upregulate P73, we examined messenger RNA (mRNA) levels of these genes in vitro following the application of the GC60 formulations of siRNA-ITCH using both RT-qPCR and Western blotting experiments (Figure 1b–f). Lipofectamine 2000 and G60-scrambled siRNA formulations were used as positive and negative controls, respectively. The silencing of the ITCH gene was achieved, as demonstrated by the mRNA expression levels for both the ITCH protein and p73 tumour suppressor protein (Figure 1c,d). ITCH mRNA expression levels were reduced by 44% and 41% for Lipofectamine 2000 and GC60, respectively (Figure 1c); P73 mRNA levels were increased, when compared to the scrambled siRNA formulation, raising from undetectable (scrambled siRNA) to 23% and 29% of the GAPDH mRNA levels for GC60-siRNA-ITCH and Lipofectamine-siRNA-ITCH, respectively (Figure 1e). In general, the siRNA gene silencing mechanism involves the selective degradation of mRNA which effectively blocks translation [36]. Without ITCH gene silencing, p73 will be lost during ubiquitination [37]

Following on from the mRNA level findings, the levels of ITCH and p73 proteins were examined. The protein samples were collected from U87MG cells 2 days and 3 days post-transfection with siRNA formulations and Western blot experiments performed on the isolated proteins (Figure 1b). The protein expression was normalised to β-actin. The ITCH protein levels in U87-MG cells treated with siRNA-ITCH G60 and Lipofectamine 2000 formulations were significantly downregulated by 34% and 43%, respectively (*p* < 0.001), compared to control cells treated with the G60-scrambled siRNA (Figure 1d). In turn, p73 protein levels were also increased 3 days after transfection (Figure 1b,f). In essence, GC60-siRNA-ITCH downregulated ITCH and resulted in an upregulation of p73 in U87MG cells. There was no significant difference in ITCH and P73 protein levels when cells were treated with either GC60-siRNA-ITCH and Lipofectamine 2000-siRNA ITCH (Figure 1d,f).

Gene transfection with GC60 was also carried out in a number of cell lines (U87-MG, A549 and MDA-MB231) as shown in the supplementary information file (Figure S2 in Supplementary Information). 

### 3.3. Gene silencing In Vitro in U87MG Cells and Apoptosis

**Figure 2:**
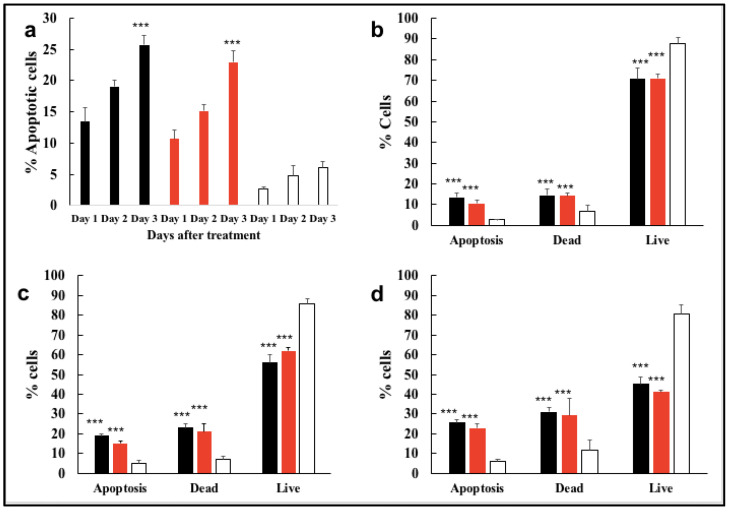
ITCH gene silencing (mean ± s.d. n = 4) resulted in an increase in U87MG cell apoptosis (**a**), as determined using flow cytometry and both Annexin V and propidium iodide (PI) responses on Days 1 (**b**), 2, (**c**) and 3 (**d**) following the application of GC60-siRNA-ITCH polyplexes (GC60, siRNA-ITCH = 100:1 g/g). Lipofectamine 2000-siRNA-ITCH lipoplexes (Lipofectamine 2000, siRNA = 2:1 g/g) and scrambled siRNA polyplexes (prepared in exactly the same way as the siRNA-ITCH polyplexes) were used as the positive and negative control formulations, respectively. (**a**) Analysis for apoptotic (Annexin V–positively stained populations) and dead (PI–positively stained populations) on Days 1, 2 and 3 showed a significant increase in apoptotic and dead cells, when siRNA-ITCH formulations were applied, when compared to the application of scrambled siRNA formulations (black bars = Lipofectamine 2000, siRNA-ITCH = 2:1 g/g, red bars = GC60, siRNA-ITCH = 100:1 g/g, white bars = GC60, siRNA-scrambled = 100:1 g/g). (**b**–**d**) Analysis for apoptotic and dead cells on Days 1, 2 and 3, respectively (black bars = Lipofectamine 2000, siRNA-ITCH = 2:1 g/g, red bars = GC60, siRNA-ITCH = 100:1 g/g, white bars = GC60, siRNA-scrambled = 100:1 g/g). There were no significant (ns) differences in the population of apoptotic or dead cells when siRNA-ITCH was applied as either a GC60 or a Lipofectamine 2000 formulation. *** = *p* < 0.001, when compared to the relevant control treated cells. The experiment was repeated four times.

The application of GC60-siRNA-ITCH polyplexes resulted in reduced cell proliferation in U87MG cells (Supplementary Information—Figure S3), reducing cell proliferation by about 45% when compared to control cells treated with GC60-scrambled siRNA polyplexes. The mechanism of anti-proliferation was then probed using flow cytometry and the AlexaFluor^TM^ 488 conjugate (Annexin V) in combination with the live/dead dye (PI) in order to quantify early apoptotic (active) and late-stage apoptotic (dead) cell populations. The results illustrate a significantly higher proportion of apoptotic (Annexin V-positively stained) and dead (PI–positively stained) cell populations in cells treated with siRNA-ITCH either as a GC60 or Lipofectamine 2000 formulation, when compared to cell populations treated with scrambled siRNA (Figure 2). With GC60-siRNA-ITCH polyplexes, the proportion of apoptotic cells increased from Day 1 (11%) to Day 3 (23%) and the proportion of apoptotic plus dead cells increased from Day 1 (25%) to Day 3 (60%, *p* < 0.001, Figure 2). With Lipofectamine 2000-siRNA-ITCH lipoplexes, the proportion of apoptotic cells increased from Day 1 (15%) to Day 3 (26%) and the proportion of apoptotic plus dead cells increased from Day 1 (25%) to Day 3 (65%, *p* < 0.001, Figure 2).

In contrast, the proportion of apoptotic cells seen when cells were treated with GC60-scrambled siRNA formulations did not increase from Day 1 (3%) to Day 3 (4%, *p* > 0.050, Figure 2). There was no significant difference in the apoptosis/dead cell populations when cells were treated with siRNA-ITCH in the form of GC60 polyplexes or Lipofectamine 2000 lipoplexes, but there were significant differences in the apoptotic/dead populations when the siRNA-ITCH treated cells were compared to the scrambled siRNA treated cell controls (Figure 2c,d, *p* < 0.0001). 

**Figure 3:**
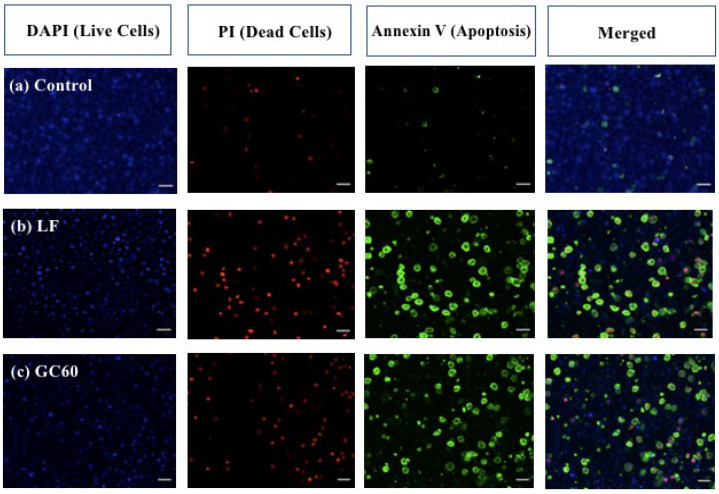
ITCH knockdown in U87MG cells led to apoptosis; cells were dosed with G60-siRNA-ITCH (GC60, siRNA = 100:1 g/g, 20 μg/mL siRNA, GC60) or with Lipofectamine 2000-siRNA-ITCH (Lipofectamine 2000, siRNA = 2:1 g/g, 20 μg/mL siRNA, LF) or with GC60-scrambeld siRNA prepared in exactly the same manner as GC60-siRNA-ITCH, 20 μg/mL siRNA, Control). Cells were visualized after incubation with the formulations for 24 h and stained as described in the Methods Section with Annexin V, DAPI and PI. PI was visualised by the red signal, Annexin V was visualised by the green signal and the cell nucleus was visualised by the blue signal, scale bar = 50 µm. The experiment was repeated four times.

To further assess the effect of GC60-siRNA-ITCH, confocal microscopy (Figure 3) was used to visualise the apoptotic and dead cell populations on application of the GC60-siRNA-ITCH to U87MG cells, when compared to the positive control (Lipofectamine 2000-siRNA-ITCH) and the negative control (GC60-scrambed siRNA). An increase in the population of apoptotic cells (stained green with Annexin V) and dead cells (stained red with PI) were observed when cells were treated with siRNA-ITCH formulations (Figure 3b,c), when compared with the negative control cells treated with scrambled siRNA (Figure 3a). A decrease in live cells (stained blue with DAPI) was also observed in the cells treated with siRNA-ITCH formulations when compared to the cells treated with the negative control, scrambled siRNA formulation (Figure 3). These data (Figure 3) present qualitative evidence that ITCH knockdown (Figure 1b–f and Figure 2) led to an increase in the population of apoptotic and dead cells, with a concomitant reduction in the population of live cells. Additional data are given in Figure S3b of the Supplementary Information. We conclude that the silencing of the ITCH gene leads to an upregulation of P73 and an increase in the population of apoptotic cells. The evidence suggests that the ITCH gene is implicated in the dysregulation seen in these immortalised cell lines.

### 3.4. Cytotoxicity Studies

**Figure 4:**
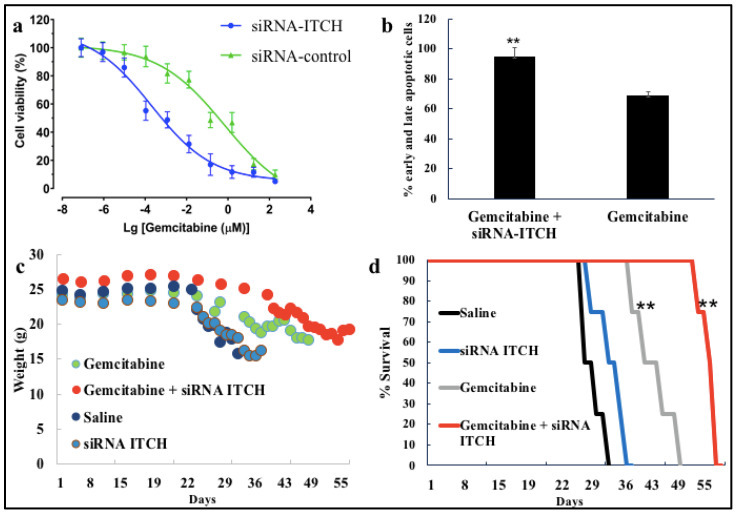
Gemcitabine chemosensitisation using siRNA-ITCH. (**a**) The cytotoxicity of gemcitabine in U87MG cells in the presence of GC60-siRNA-ITCH (GC60, siRNA = 100:1 g/g, 8 μg/mL siRNA) and GC60-siRNA-scrambled prepared in exactly the same manner and dosed at exactly the same dose as the siRNA-ITCH formulation, gemcitabine IC50 (siRNA-ITCH) = 0.02 μM, gemcitabine IC50 (siRNA-scrambled) = 0.68 μM (mean ± s.d. n = 4 wells on 4 different separate occasions, *p* < 0.001). (**b**) Gemcitabine-induced apoptosis in U87MG cells is enhanced in the presence of GC60-siRNA-ITCH (GC60 siRNA = 100:1 g/g, 20 μg/mL siRNA, 10μg/well) when compared to GC60-siRNA-scrambled, ** = *p*< 0.01, (gemcitabine concentration = 50 mg/mL^−1^, mean ± s.d., n = 4, repeated on 4 separate occasions). (**c**) The body weights of CD-1 nude mice bearing U87MG-Luc2 tumours showing the decline in body weight as the tumour develops within the different groups. Mice were dosed with siRNA formulations at 0.081 mg/kg and with gemcitabine at 33 mg/kg as described in the methods. (n = 4 mice per group) (**d**) Kaplan–Meier survival analysis for CD-1 nude mice bearing U87MG-Luc2 tumours with the graph showing survival (from tumour implantation to time of euthanasia as a humane end point) of the different groups of animals, with animals receiving siRNA formulations at 0.081 mg/kg and with gemcitabine at 33 mg/kg as described in the Methods Section, n = 4 mice each group, ** = *p* < 0.01, when compared to non-treated animals.

Cytotoxicity studies with gemcitabine in the presence of GC60-siRNA-ITCH show that siRNA-ITCH clearly increased the efficacy of gemcitabine, decreasing the IC50 from 0.68 μM (siRNA-scrambled) to 0.02 μM (siRNA-ITCH), and in effect, demonstrating chemosensitisation of the drug (Figure 4).

### 3.5. In Vivo Tumouricidal Activity

**Figure 5:**
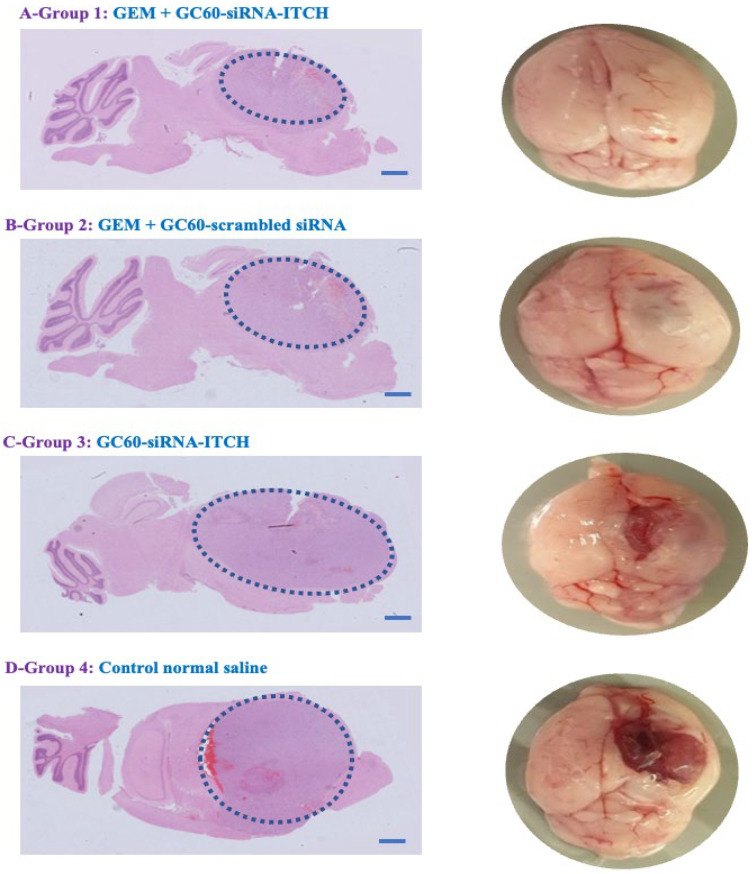
Haematoxylin and eosin (H&E)-stained brain samples, showing tumour growth in animals culled on Day 54 (GC60-siRNA-ITCH and gemcitabine), Day 45 (GC60-siRNA-scrambled and gemcitabine), Day 31 (GC60-siRNA-ITCH) and Day 29 (normal saline). Dashed blue line delineates the tumour on the image.

Following on from the in vitro cytotoxicity observed (Figure 4a,b), tumouricidal activity was seen in mice bearing the U87MG-Luc tumours (Figure 4c,d) when dosed with a combination of GC60-siRNA-ITCH (0.081 mg/kg siRNA-ITCH) daily from Day 4—Day 7 after tumour implantation and gemcitabine (33 mg/kg) on Days 4, 7 and 10 (Figure 4c,d; Figure 5 and Figure S4 in the Supplementary Information). 

The group treated with GC60-siRNA-ITCH (0.081 mg/kg siRNA) in combination with gemcitabine (33 mg/kg) survived the longest (Figure 4d) when compared to all other groups (** *p* < 0.01), with a mean survival time of 54 days, while mice treated with gemcitabine alone (33.3 mg/kg) had a mean survival time of 45 days (** *p* < 0.01). The control group (normal saline) and the GC60-siRNA-ITCH only group had mean survival times of 29 days and 33 days, respectively (Figure 4). The combination treatment of siRNA-ITCH and gemcitabine extended survival by 89% when compared to animals receiving no treatment. It is important to note that while the IVIS imaging (Figure S5 in the Supplementary Information) did not show a discernible difference in tumour growth on Day 25, post tumour implantation, the H&E-stained histology samples (Figure 5 and Figure S4 in the Supplementary Information) did show that the GC60-siRNA-ITCH/gemcitabine treated mice had a lower tumour burden. There appeared to be no difference in size when tumours of mice dosed with normal saline were compared to those of mice dosed with GC60-siRNA-ITCH alone. 

These data (Figure 4 and Figure 5) suggest that siRNA-ITCH enhanced the efficacy of gemcitabine, effectively chemosensitising the drug and, thus, significantly prolonging the survival of CD-1 nude mice bearing U87MG-Luc2 tumours. In vivo evidence of ITCH downregulation and P73 upregulation was also observed (Figure S6 in the Supplementary Information).

### 3.6. Histological Gross Toxicology

**Figure 6:**
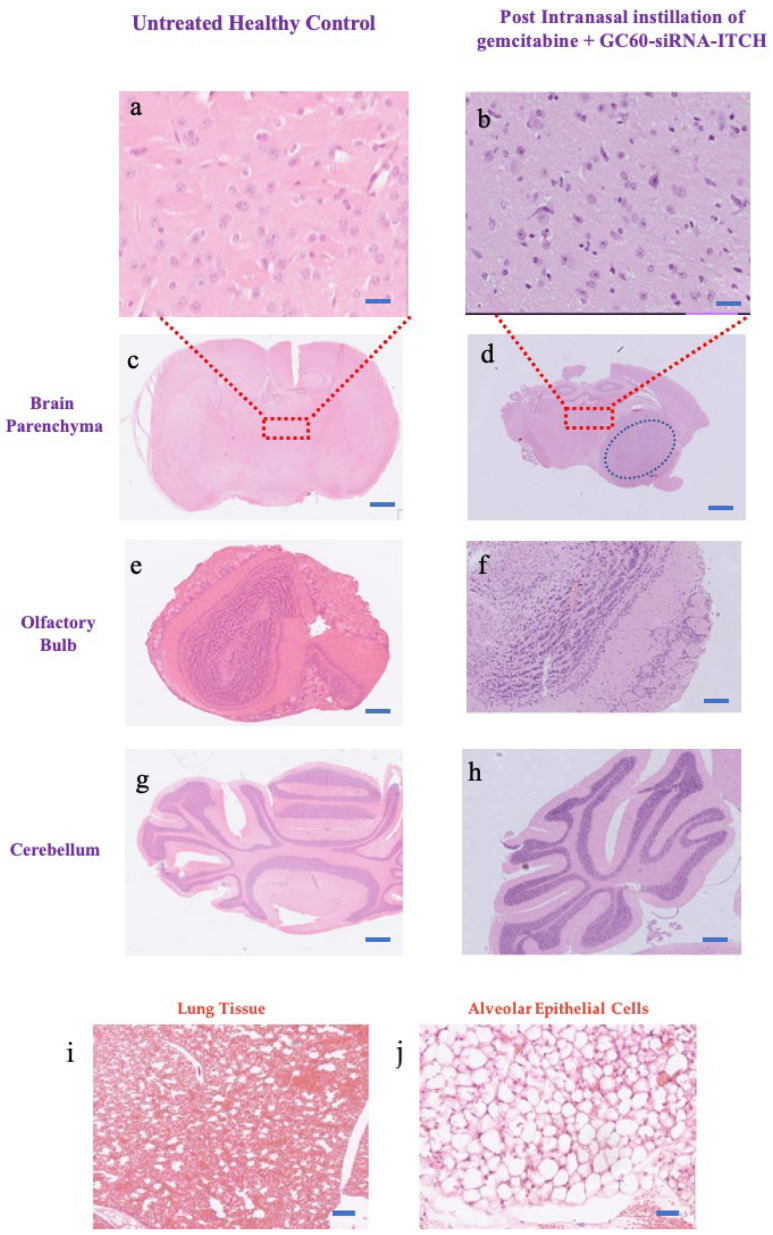
Absence of gross histological olfactory bulb and parenchymal tissue toxicity in treated and untreated CD-1 nude mice bearing the U87 MG-Luc2 tumour. The H&E-stained tissues illustrate no damage to (**a**–**d**) brain parenchyma (**a**,**b**), as can be seen in the magnified images of the brain parenchyma that show no clustering of the neurons and astrocytes and no sign of haemorrhage or other tissue damage, (**e**,**f**) olfactory bulb, (**g**,**h**) cerebellum, (**i**,**j**) lungs and alveoli, in the group treated via the nose to brain route with the combination of GC60-siRNA-ITCH (6 doses of 0.081 mg/kg siRNA-ITCH) and gemcitabine (3 doses of 33 mg/kg) when compared to the untreated control group of the CD-1 nude mice. The blue dashed line delineates the intracranial tumour. Scale bar = 2 mm for whole tissue images, 1 mm for the treated olfactory bulb tissue image and 100 μm for brain parenchyma images.

The histological examination with H&E staining (Figure 6 and Figure S5 in the Supplementary Information) on both the untreated and the treated animals was conducted with specific emphasis on the following regions: olfactory bulb tissues, brain parenchymal tissues and lungs. The examination identified no evidence of tissue damage, inflammation, haemorrhage or gross toxicity as a result of treatment with gemcitabine (concentrations of 33.3 mg/kg on day 4, 7 and 10) and GC60-siRNA-ITCH (0.081 mg/kg on days 4–10). The high-power photomicrographs display a typical cellular structure (glial cells and neurons) in the brain parenchyma of the untreated group (Figure 6a) and the group treated with the combination of GC60-siRNA-ITCH and gemcitabine (Figure 6b).

## 4. Discussion

In this study, we explore a new therapeutic approach for the treatment of intracranial tumours (Figure 4). The treatment is based on the delivery of siRNA-ITCH to silence the expression of the ITCH ligase protein (Figure 1) and thus reduce the ubiquitination of and thus the degradation of p73 protein (Figure 1). The decreased degradation of p73 leads to increased levels of p73 and a pro-apoptotic shift of the cellular homeostasis (Figure 2 and Figure 3), which amplifies the apoptotic effects of gemcitabine (Figure 4a,b). We have previously shown this approach to work in flank tumour pancreatic cancer xenografts [17]; others have used this approach in experimental lung cancer xenografts [38], and sensitisation to radiation has been shown in neuroblastoma cells, as has siRNA ITCH knockdown in neuroblastoma xenografts in vivo [39]. However, this is the first time that this combined siRNA–chemotherapy approach has been shown to be beneficial in the treatment of intracranial tumours. The nose to brain route of administration was critical to the success of the approach and this is the first demonstration of this therapeutic effect when using the nose-to-brain route. The data validate the ITCH gene as a therapeutic target as although the silencing of the ITCH gene alone had a non-significant effect on tumour bearing animal survival, the combination of ITCH gene silencing and the delivery of gemcitabine provided a demonstrable therapeutic effect with survival extended by 89% when compared to control untreated animals (Figure 4d and Figure 5 and Figure S6 in the Supplementary Information). No gross toxic effects were observed with respect to animal weights (Figure 4c), the brain histology samples and the more distal organ histology samples (Figure S7—Supplementary Information). 

Gemcitabine, a nucleoside, inhibits DNA synthesis [21,22,23] and the inhibition of DNA synthesis, occurs via the incorporation of gemcitabine monophosphate into DNA, which is followed by the formation of large-size DNA fragments and the triggering of gemcitabine-induced apoptosis [40]. The large sized DNA fragments were observed in human leukaemia cells (CEM) [40]. In pancreatic cancer cells (PANC-1) [41], specific genes have been identified as being associated with gemcitabine-induced apoptosis. In PANC-1 cells, gemcitabine promotes cellular apoptosis by downregulating the antiapoptotic gene, *PAP*, and upregulating the pro-apoptotic *TP53INP1* gene and GSK-3β^ser9^ protein [41]. Reduced function of p53 is a common cause of cancer therapy resistance; p53 is functionally deficient in nearly 50% of cancers and is linked to reduced drug sensitivity and poorer survival outcomes [42]. In p53 deficient cells, gemcitabine works via p73, which acts as a functional analogue of p53 [43]. ITCH is an E3 ligase that is overexpressed in different cancers and plays a critical role in inhibiting and degrading P73, a tumour suppressor gene [44]. Targeting the ITCH gene is a promising therapeutic strategy for treating different types of cancer and improving chemosensitivity [17,45,46]. Silencing the ITCH gene contributes synergistically to the apoptosis seen in U87MG cells on treatment with gemcitabine (Figure 4b), and while we did not determine the level of apoptosis at the target tumour area in vivo, the efficacy of the combined effect of ITCH gene silencing (Figure S6—Supplementary Information) and gemcitabine delivery on the target tumour tissue is demonstrated by an increase in animal survival (Figure 4d) and a decrease in tumour burden (Figure 5). The combination treatment was well tolerated for the first 30 days before animals began to succumb to the tumour burden (Figure 4c). Our results are in line with those of Li and Zhang, who discovered an elevated ITCH expression in lung cancer tissues [38]. Their study also demonstrated the effectiveness of siRNA-ITCH alone in promoting cellular apoptosis and inhibiting the invasion and proliferation of lung cancer cells via regulation of the Bcl2/Bax, EMT and MMP signalling pathways [38]. Tumour suppressor genes are usually mutated in different cancers, which leads to an uncontrolled cell cycle and, thereby, uncontrolled proliferation [47]. Hence, restoring function to the tumour suppressor gene family, including p73 and p53, is a valid therapeutic target. 

Meng et al. found that the downregulation of ITCH by nanoencapsulated siRNA-ITCH is comparatively slow [39]. On Day 1 after transfection, ITCH was not silenced in either in vitro or in vivo experiments; however, ITCH silencing was detected at Day 2 post-transfection [39]. Our results showed a similar tendency of slow ITCH gene silencing (Figure 1). On Day 1 post-transfection with GC60-siRNA-ITCH and with Lipofectamine 2000-siRNA-ITCH, ITCH gene silencing was not detected in U87MG cells. However, ITCH gene silencing was detected on Day 2 post-transfection, with GC60-siRNA-ITCH and with Lipofectamine 2000-siRNA-ITCH, with significantly decreased levels of ITCH expression detected (Figure 1). Control formulations with scrambled siRNA showed no gene silencing (Figure 1). Apoptosis also increased on Day 3 post transfection when compared to Day 1 post transfection (Figure 2) when cells were treated with GC60-siRNA-ITCH and with Lipofectamine 2000-siRNA-ITCH. It is worth noting that the nose-to-brain delivery of gemcitabine alone also resulted in a significant tumouricidal effect (Figure 4c and Figure 5). Gemcitabine is a nucleoside analogue that possesses broad-spectrum anti-tumour activity [30,48]. However, as well as intrinsic resistance to gemcitabine [25], there is also limited penetration of gemcitabine into solid tumours [30,31,32] and the drug has a short plasma half-life due to degradation by cytidine deaminase in the circulation [28]. All of these factors limit the effectiveness of parenteral gemcitabine. Different invasive strategies have been applied to deliver gemcitabine directly to brain tumours. For example, Diegen et al. [49] delivered gemcitabine directly to the CNS of rats bearing a 9 L glioma by convection-enhanced delivery (CED), a bulk-flow mediated invasive method that delivers drugs through the interstitial spaces of the brain parenchyma [49]. In contrast to this previous very invasive method of drug administration offered by others, we found that the nose-to-brain delivery of gemcitabine to a U87-MG-Luc2 model at three doses (at 3-day intervals) of 33 mg/kg, resulted in significant tumoricidal activity (Figure 4c).This dosing regimen was more efficient than the daily intraperitoneal injections reported in several preclinical models [48,50,51,52]. This is the first report of intranasal gemcitabine demonstrating a significant tumouricidal response, and it is possible that this route of administration may lead to higher response rates in humans compared to the use of the intravenous route [53] of administration.

Interestingly, silencing ITCH alone did not result in tumouricidal activity and a tumouricidal response required the additional administration of gemcitabine (Figure 4c). The ITCH gene was not permanently knocked down and we speculate that this could be the reason for the low level of tumouricidal activity seen in animals administered GC60-siRNA-ITCH alone. Our previous studies with shRNA-ITCH (small hairpin RNA-ITCH—delivered as a plasmid) and a pancreatic cancer flank xenograft did not reveal the superiority of gemcitabine alone vs control untreated samples [17].

In conclusion, our data indicate that a combination of GC60-siRNA-ITCH and gemcitabine, delivered via the nose-to-brain route, may be used to treat intracranial tumours. Glioblastoma multiforme (GBM) is a fast-growing and aggressive brain tumour, classified as a grade IV astrocytoma [39,54]. Despite best efforts to develop therapeutic strategies for GBM, including surgery, chemotherapy, radiation or combination therapies, the prognosis is still poor for GBM patients, with a median survival time of about 12–15 months [55,56,57]. These data provide a motivation to trial nose-to-brain chemotherapy and gene silencing treatments in human tumours. 

## Data Availability

The data supporting the findings of the study are available from the corresponding author, upon reasonable request.

## Supplementary Materials

The following supporting information can be downloaded at: https://www.mdpi.com/article/10.3390/pharmaceutics16060829/s1. Figure S1: The clinical and distress scores sheet; Figure S2: In vitro transfection in various cell lines with pDNA GFP, (a–c) fluorescent images of different cell lines, cells were dosed with G60-pDNA-GFP (GC60, pDNA = 100: 1 g/g, 10 μg/mL pDNA) and cells were visualized using EVOS Fluorescence Microscopy, after incubation with the formulations for 48 h, bar = 400 nm; (d–f) Luciferase gene expression in different cell lines, cells were dosed with G60-pDNA-Luc (GC60, pDNA = 100:1 g/g, 10 μg/mL pDNA) or Lipofectamine 2000–pDNA-Luc (Lipofectamine 2000, pDNA 2:1 g/g 10 μg/mL pDNA and the unit of Luciferase gene expression was described as Relative luminescence unit (RLU); Figure S3a: The inhibition of U87-MG cell progression on knockdown of the ITCH gene following the application of GC60-siRNA-ITCH (100:1 g/g), at a dose of 0.02 mg/mL siRNA-ITCH, as illustrated by the MTT assay data, scrambled siRNA formulations were prepared in exactly the same manner as siRNA-ITCH formulations, *** *p* < 0.001. 3b: ITCH knockdown in U87MG cells led to apoptosis, cells were dosed with G60-siRNA-ITCH (GC60, siRNA = 100:1 g/g, 20 μg/mL siRNA, GC60) or with Lipofectamine 2000-siRNA-ITCH (Lipofectamine 2000, siRNA = 2:1 g/g, 20 μg/mL siRNA, LF) or with GC60-scrambeld siRNA prepared in exactly the same manner as GC60-siRNA-ITCH, 20 μg/mL siRNA, Control). Cells were visualized after incubation with the formulations for 24 h and stained as described in the Methods with Annexin V, DAPI and PI. PI was visualized by the red signal, Annexin V was visualized by the green signal, and the cell nucleus was visualized by the blue signal, scale bar = 10 µm. Figure S4: Representative images of H&E stained histology samples of different brain sections of CD-1 nude mice bearing U87-MG-Luc2 xenograft taken on Day 30 after tumour implantation: (a) dosed with gemcitabine (33 mg/kg on Days 4, 7 and 10) in combination with GC60–siRNA-ITCH (siRNA, GC60 ratio = 1:100 g/g) and dosed with 0.081 mg/kg siRNA daily on Days 4 to 10 inclusive, (b) dosed with gemcitabine (33 mg/kg on Days 4, 7 and 10) plus GC60–scrambled siRNA (prepared and dosed in exactly the same way as the siRNA-ITCH formulations), (c) dosed with GC60–siRNA-ITCH (siRNA, GC60 ratio = 1: 100 g/g) and dosed with 0.081 mg/kg siRNA daily on Days 4 to 10 inclusive, (d) dosed with normal saline (0.03 mL daily). Scale bar = 2 mm. Figure S5: Representative IVIS luminescence intensity images taken at Day 25 post-tumour implantation for 4 groups of animals (n = 4) intranasally dosed with various formulations: Group 1 dosed with normal saline (0.030 mL daily), Group 2 dosed with GC60 – siRNA-ITCH (siRNA, GC60 ratio = 1: 100 g/g) and dosed with 0.081 mg/kg siRNA daily on Days 4 to 10 inclusive, Group 3 dosed with gemcitabine (33 mg/kg on Days 4, 7 and 10) plus GC60–scrambled siRNA (prepared and dosed in exactly the same way as the siRNA-ITCH formulations), Group 4 dosed with gemcitabine (33 mg/kg on Days 4, 7 and 10) in combination with GC60–siRNA-ITCH (siRNA, GC60 ratio = 1:100 g/g) and dosed with 0.081 mg/kg siRNA daily on Days 4 to 10 inclusive. Figure S6: In vivo brain gene silencing showing down regulation of the ITCH protein from Western Blot data, as described in the Methods above. Figure S7: Raw Western Blot data showing the in vitro gene silencing in the U87-MG cell line. Down regulation of ITCH and the upregulation of p73 is seen and the methods are described above. Figure S8: Absence of gross histological olfactory bulb and parenchymal tissue toxicity in CD-1 nude mice bearing the U87 MG-Luc2 group treated via the nose to brain route with the combination of GC60-siRNA-ITCH (6 doses of 0.081 mg/kg siRNA-ITCH) and gemcitabine (3 doses of 33 mg/kg). Scale bar = 2 mm for whole tissue and 100 μm for brain parenchyma images.
