# Supplementary material for: Targeting Intracranial Tumours with a Combination of RNA and Chemotherapy
Source: Pharmaceutics. 2024 Jun 18;16(6):829. doi: 10.3390/pharmaceutics16060829 (PMC11207522; doi:10.3390/pharmaceutics16060829)

Supplementary Information

Targeting of mouse intracranial tumours with a combination of RNA and chemotherapy

Abdulhamid Fatani[1], Andreas G. Schatzlein[1,2], Ijeoma F. Uchegbu[1,2]

[1]UCL School of Pharmacy, 29 – 39 Brunswick Square, London WC1N 1AX

[2]Nanomerics Ltd. Nanomerics Ltd., Block Y, Northwick Park and St Mark's Hospital, Watford Road, Harrow HA1 3UJ, UK

Methods

Gene Transfection in vitro

U87-MG cells were also transfected with both green fluorescent protein and luciferase plasmids.

Luciferase:

6 - well plates were seeded with 500,000 cells (U87-MG) per well and incubated *for* 48 h. The polyplexes were prepared to a final Luciferase DNA concentration of 10 µg/mL. The polymer stock solution was prepared in sodium triphosphate buffer (20 mM, pH = 6.8) to give a polymer: DNA weight ratio of 100:1. The polyplexes were incubated at 4 °C, for 24 h, after which 0.5 mL of the polyplex dispersion was added to the cells (10 µg/mL of plasmid DNA, 5µg DNA per well) and incubated for 24 h in 1.5 mL of serum free Minimum Essential Medium (EMEM, Life Technologies, Paisley, UK) in a total volume of 2 mL per well. After 24 hours, the medium was removed from the transfected wells and replaced with fresh EMEM (2 mL per well) and the cells replenished with fresh MEM. The cells were incubated for a further 24 h. The transfection efficiency was investigated by

measuring the activity of the luciferase enzyme using the Luciferase Reporter Assay following the manufacturer's instructions (Promega, Madison, USA).

Cells were lysed by adding 400 µL of Reporter Lysis Buffer (RLB) (diluted) per well in a 6-well plate and incubating the cells at room temperature for 15 min using a plate shaker (Infors HT Minitron company, Surrey, UK). The plates well were scraped to detach the cells manually using a scraper and the lysates were transferred by pipetting into 1mL Eppendorf tube and the samples were placed on ice. The tubes were then centrifuged for 3 min at 4°C at 12,000 rpm (MIKRO 200 Microliter Tube Package 24 Micro Centrifuge, Hettich, Kirchlengern, Germany). The supernatants were transferred to fresh tubes and these lysates immediately assayed or stored at – 80°C until analysis could be performed. The Luciferase Assay Reagent (100 µL) was dispensed into each well (one well per sample) in laminator Corning™ 96-Well Solid White Polystyrene Microplates (Corning™ New York, U.S.). Following this, an aliquot (50 µL) of the sample lysate was added to the Luciferase Assay Reagent treated-well.

The samples in all wells were mixed by pipetting 2-3 times. Finally, the Corning™ 96-Well Solid White Polystyrene Microplates (Corning™ New York, U.S.) were placed in the ELx808 microplate reader (Bio-tek instruments, Potton, UK) and the readings were obtained. Values were expressed in relative luminance units (RLU) per well and compared to the data observed with the GC polymer alone, naked plasmid DNA, the positive control Lipofectamine 2,000 and the negative controls (untreated cells). The Lipofectamine 2000 polyplexes were used as a positive control and prepared at a ratio of 5 µg of DNA to 10 µg of Lipofectamine 2,000 per well and prepared based on the manufacturer's instructions. Cells were incubated for 24 h with the Lipofectamine – DNA polyplexes (5 µg DNA per well) along with 10% of serum (FBS) in media. The lipoplexes were then replaced with fresh MEM media and the cells incubated for a further 24h. Experiments were performed in triplicate and repeated three times.

GFP

6 - well plates were seeded with 500,000 cells (U87-MG) per well and incubated 48 h. The polyplexes were prepared to a final GFP -DNA concentration of *10* µg/mL. *The* polymer stock solution was prepared in sodium triphosphate buffer (20 mM, pH = 6.8) to give a polymer: DNA weight ratio of 100. The polyplex dispersion *was* incubated at 4 **°C**, for 24 h, after which 0.5 mL of the polyplex dispersion *was* added to the cells *(10* µg/mL, 5 µg per well) and incubated for 24 h in 1.5 mL of serum free Minimum Essential Medium (EMEM, Life Technologies, Paisley, UK) in a total volume of 2 mL per well. After 24 hours, the medium was removed from the transfected wells and replaced with fresh EMEM (2 mL per well) and the cells replenished with fresh MEM. The cells were incubated for a further 24 h. The transfection efficiency was Imaged by the EVOS florescence microscopy images and the scale bar is 400 µm. The same procedures were used to transfect A549 and MDA-MB231 cells.

siRNA effect on cell proliferation

The U87-MG cells were seeded at a density of 5,000 cells/well in a 96-well plate and left to recover for 48 h before being incubated with siRNA anti-*ITCH* (8 µg/mL). Then, the treatments were discarded after 24 hours, and the cells were replenished with 200 µL of serum-rich medium. This was followed by a measurement of cell viability at various time points: 24 h after recovery, 48 h after recovery and 72 h after recovery. Cell viability was measured by adding 200 µL of MTT-containing media (5 mg/mL of the MTT reagent) to each of the 96 wells and incubating the cells at 37 °C for 2 h. The media containing MTT solution was then discarded and replaced with dimethyl sulfoxide (DMSO) to lyse the cells, and the 96-well plate was shaken at room temperature for 15 min (Infors HT Minitron company, Surrey, UK). The absorbance was measured at 570 nm using an ELx808 absorbance microplate reader (BioTek Instruments, Potton, UK). The cell viability was then calculated based on the this equation : $\frac{\textbf{Absorbance of treated cells}}{\textbf{Absorbance of untreated cells}}\ \mathbf{X\ 100}$

In vivo protein expression

ITCH tumour down-regulation ***and up regulation of 73 protein expression in vivo***

The micro-dissected tissues of brain tumours were homogenised using a glass homogeniser (VWR International, Leicestershire, UK) , mixed with a T-PER tissue lysis buffer reagent supplemented with an EDTA-free Halt Protease Inhibitor Cocktail 100 × (1 ml in 100 ml of T-PER reagent). According to the manufacturer's instructions, 20 µl of T-PER reagent was added to 1 mg of tissue (100 mg of tissue in 2 mL of T-PER). The homogenates were centrifuged at 14,000 rpm for 6 min at 4°C (MIKRO 200 Microliter Tube Package 24 Micro Centrifuge, Hettich, Kirchlengern, Germany). The supernatants were transferred to fresh tubes, immediately assayed, or stored at -80°C until analysis could be performed.

Protein content from the homogenised tissues was quantified using a Bicinchoninic acid assay (BCA), and a bovine serum albumin (BSA) standard curve used (prepared between 0.056 – 2 mg/ mL). The BCA method for protein quantification was carried out by taking aliquots (2 µL) of BSA standards or suitably diluted protein samples and adding these to 200 µl water in Eppendorf tubes. The BCA reagent (200 µL) was then added to each sample and mixed well. Once mixed, samples were incubated for 5-10 minutes at room temperature. Aliquots (200 µL) of each sample were then transferred to 96-well plates and absorbance was measured at a wavelength of 595nm in a plate reader (PHERAstar, BMG LABTECH, Ortenberg, Germany). The unknown protein concentrations were calculated using the linear equation derived from the calibration curve.

Gels were run at 100 V in a tank buffer (sodium dodecyl sulphate (0.1%), Glycine (192 mM) in Tris buffer (25 mM), pH = 8.3) for 1 h or at least until the bromophenol blue dye front reached the bottom of the gel. Tris buffer consists of: NaCl (150 mM) and Tris (20 mM) made to the required pH with NaOH (0.1M) or HCL (0.1M). Once electrophoresis was completed, the gel was carefully removed from the cassette and transferred onto a nitrocellulose membrane (0.45 µm; Bio-Rad). A typical sponge–paper–membrane–gel–paper–sponge transfer sandwich was assembled to transfer proteins.

The sponges, blotting papers and membrane were soaked in the transfer buffer for at least 10 minutes prior to the assembly of the transfer sandwich. Blotting took place in the voltaged tank filled with the cold transfer buffer (glycine (192 mM), Tris (25 mM) all in 20% v/v methanol, pH = 8.3) for 75 min at 100 V.

Membranes were then incubated with blocking buffer containing Tris-buffered saline with Tween 20 (0.1%w/v), pH = 7.4, (TBST) and containing 5% non-fat milk, for 1 h at room temperature. After that, the membranes were incubated separately with the primary mouse monoclonal antibodies against ITCH (at 1:1,000 dilution) Thermo Fisher, Invitrogen (Loughborough, UK), P73 (at 1:1,000 dilution) Thermo Fisher, Invitrogen (Loughborough, UK) and actin (at 1:1,000 dilution) Thermo Fisher, Invitrogen (Loughborough, UK) at 4 °C and overnight. All dilutions were carried out in TBST, pH = 7.4. The membranes were subsequently washed with TBST (pH = 7.4) three times and then, incubated with mouse anti-human IgG secondary antibodies (at 1:1, 000 dilution) conjugated with horseradish peroxidase (HRP) at room temperature for 1 h. The membranes were then washed three times with TBST buffer (pH = 7.4). A SuperSignal™ West Femto Maximum Sensitivity chemiluminescent substrate kit (Thermo Fisher Scientific, Oxford, UK) was used to catalyse HRP with luminol to generate luminescence. Then the membrane was imaged by a ChemiDoc™ MP system (Bio-Rad, Watford, UK) and analysed using Image Lab software (Bio-Rad).

Normalization of total protein concentration in the samples

A small volume of the supernatant of the homogenized brain tumor tissues was taken to perform protein estimation assay. The protein concentration of unknown samples was determined by comparison with the standards, and the standard is diluted into the same buffer as the unknown samples. Protein estimation was performed using BCA assay (as detailed above), absorbance at 280 nm. After that, an appropriate volume of lysates was transferred to microcentrifuge tubes so that all samples contained the same total protein concentration. Finally, an adequate ice-cold lysis buffer was

added to make up all the lysates to the same volume. Data were generated as gel blots images. Furthermore, the ITCH and P73 relative expression levels were normalised to the reference protein actin to correct for sample variation. The ratio of ITCH and P3 expression to actin expression was determined and ITCH expression was compared between samples. ITCH and P73 protein levels were expressed as protein expression percentage relative to controls.

Figure S1: The clinical and distress scores sheet

Distress and Condition Score Sheet

Experiment　　Start date

Group　　Treatment

Animal ID

PARAMETER		SCORE	OBSERVATION (Date/Time)	OBSERVATION (Date/Time)	OBSERVATION (Date/Time)	OBSERVATION (Date/Time)	OBSERVATION (Date/Time)	OBSERVATION (Date/Time)	OBSERVATION (Date/Time)
Appearance	Normal	0							
	General lack of grooming	1							
	Piloerection, fresh ocular and nasal discharges	2							
	Piloerection, hunched up	3							
	Above and eyes half closed	4							
Natural Behaviour	Normal	0							
	Minor changes	1							
	Less mobile and isolated, but alert	2							
	Restless or very still, not alert	3							
Hydration Status	Normal	0							
	Abnormal skin pinch test	5							
Clinical Signs	Normal respiratory rate and pattern	0							
	Slight changes, increased rate only	1							
	Increased rate with abdominal breathing	2							
	Decreased rate with abdominal breathing	3							
	Marked abdominal breathing and cyanosis	4							
Provoked Behaviour	Normal	0							
	Minor depression or exaggerated response	1							
	Moderate change in expected behaviour	2							
	Very weak and precomatose	3							
Body condition	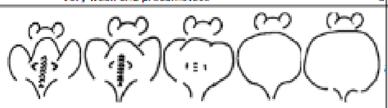 Score: 10, 5, 0, 0, 3								
		Total	0	0	0	0	0	0	0

Action	Other comments
0±4 Normal<br>5±9 Monitor carefully, consider analgesics<br>10±14 Suffering, provide relief, observe regularly. Seek second opinion from named animal care and welfare officer and/or named veterinary surgeon. Consider termination<br>15±20 Severe distress. Actual Severity Reporting	

Figure S2: ***In vitro transfection*** **in various cell lines with pDNA GFP, (a – c) fluorescent images of different cell lines, cells were dosed with G60-pDNA-GFP (GC60, pDNA = 100: 1 g/g, 10 μg/ mL pDNA) and cells were visualized using EVOS Flouresence Microscopy , after incubation with the formulations for 48 hours, bar = 400 μm; (d – f) Luciferase gene expression in different cell lines , cells were dosed with G60-pDNA-Luc (GC60, pDNA = 100: 1 g/g, 10 μg/ mL pDNA) or Lipofectamine 2000 – pDNA-Luc (Lipofectamine 2000, pDNA 2: 1 g/g 10 μg/ mL pDNA) and the unit of Luciferase gene expression was described as Relative luminescence unit (RLU) .**

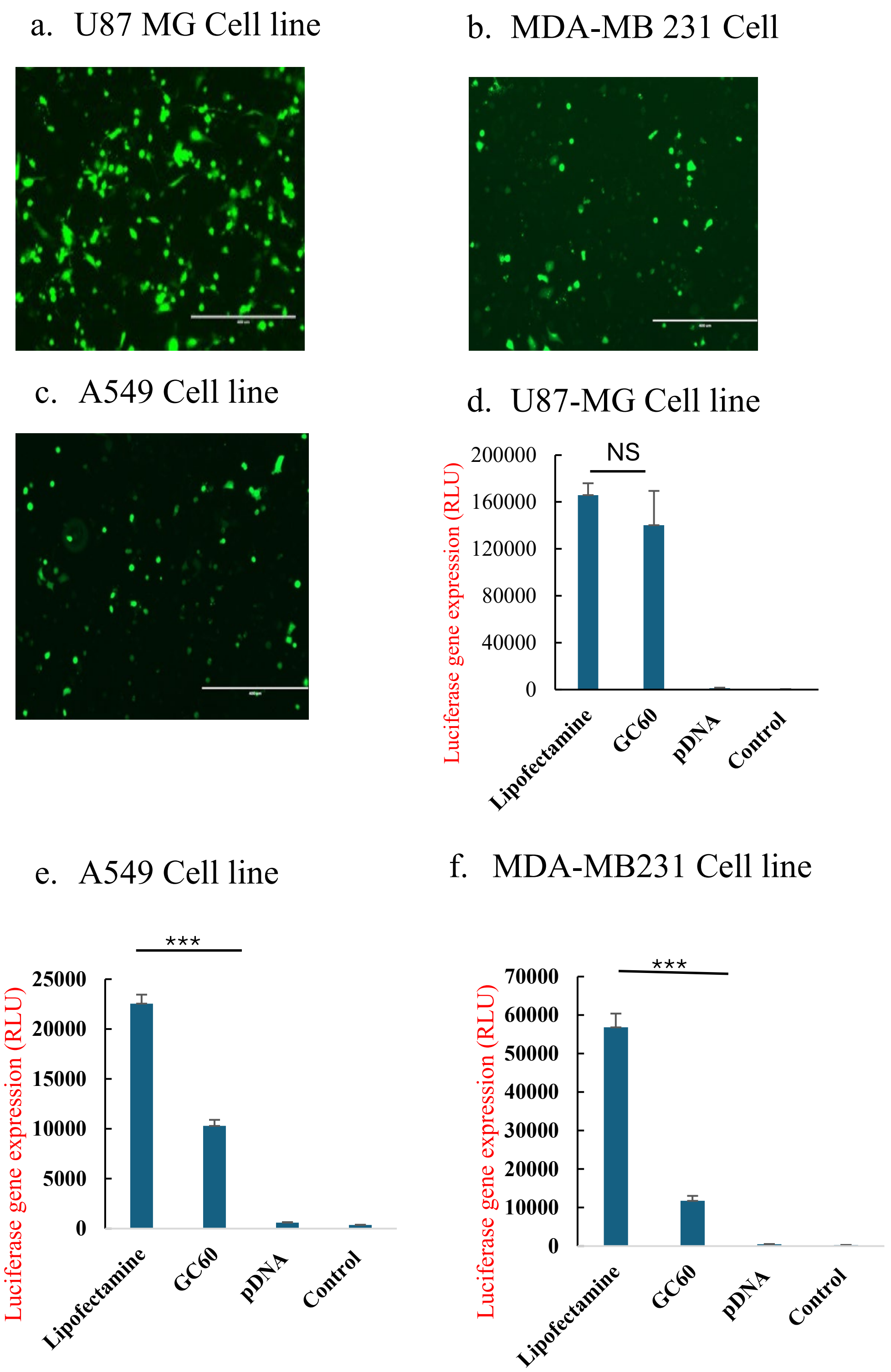
a. U87 MG Cell line
b. MDA-MB 231 Cell
c. A549 Cell line
d. U87-MG Cell line
NS
200000
160000
120000
80000
40000
0
Luciferase gene expression (RLU)
Lipofectamine
GC60
pDNA
Control
e. A549 Cell line
***
25000
20000
15000
10000
5000
0
Luciferase gene expression (RLU)
Lipofectamine
GC60
pDNA
Control
f. MDA-MB231 Cell line
***
70000
60000
50000
40000
30000
20000
10000
0
Luciferase gene expression (RLU)
Lipofectamine
GC60
pDNA
Control

Figure S3a: The inhibition of U87-MG cell progression on knockdown of the ITCH gene following the application of GC60-siRNA-ITCH (100: 1g/g), at a dose of 0.02 mg/ mL siRNA-ITCH, as illustrated by the MTT assay data, scrambled siRNA formulations were prepared in exactly the same manner as siRNA-ITCH formulations, ***p<0.001.

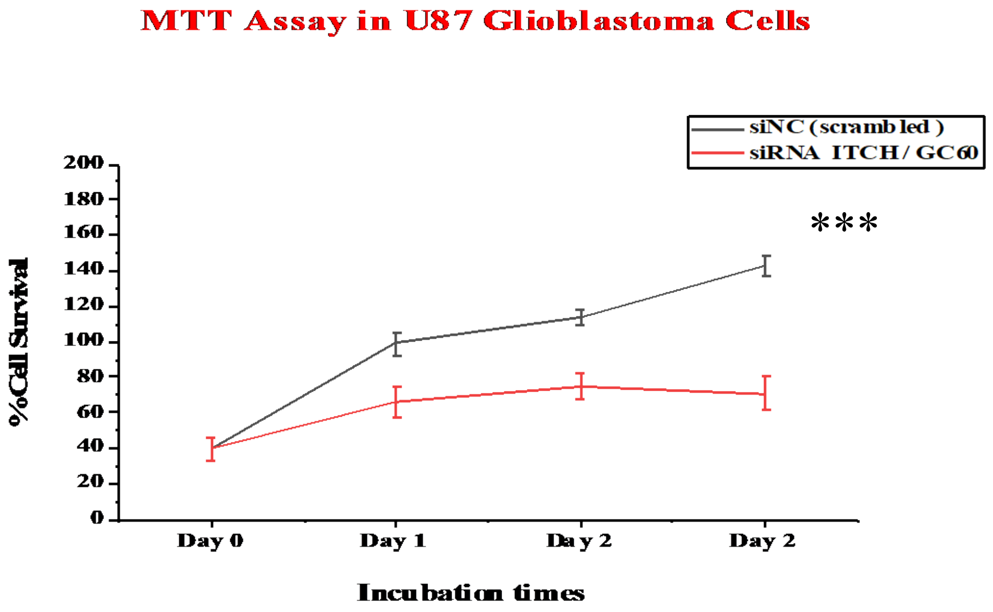

Figure S3b: ITCH knockdown in U87MG cells led to apoptosis, cells were dosed with G60-siRNA-ITCH (GC60, siRNA = 100: 1 g/g, 20 µg/ mL siRNA, GC60) or with Lipofectamine 2000-siRNA-ITCH (Lipofectamine 2000, siRNA = 2: 1 g/g, 20 µg/ mL siRNA, LF) or with GC60-scrambeld siRNA prepared in exactly the same manner as GC60-siRNA-ITCH, 20 µg/ mL siRNA, Control). Cells were visualized after incubation with the formulations for 24 hours and stained as described in the Methods with Annexin V, DAPI and PI. PI was visualized by the red signal, Annexin V was visualized by the green signal, and the cell nucleus was visualized by the blue signal, scale bar = 10 µm.

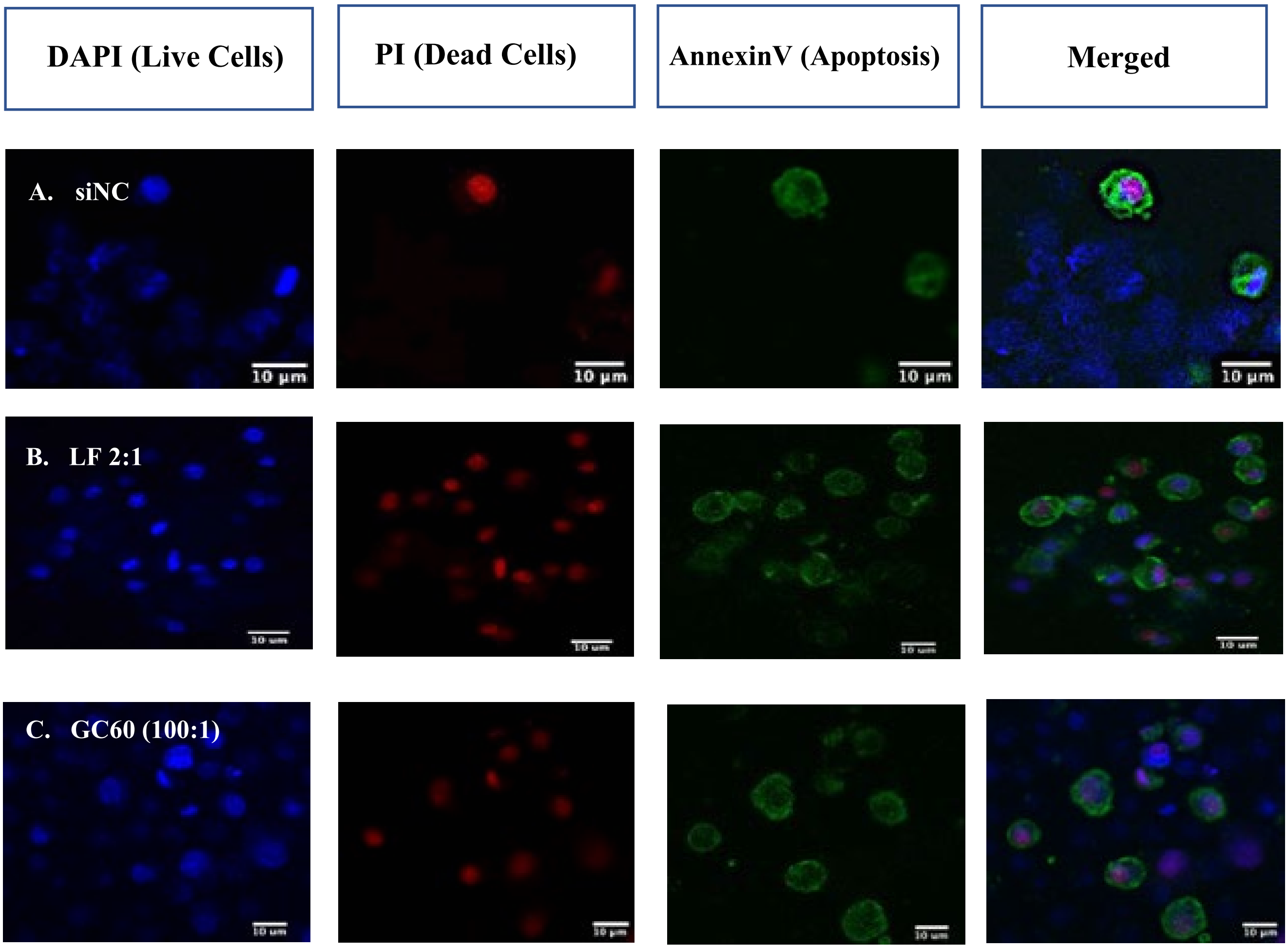

Figure S4: Representative images of H&E stained histology samples of different brain sections of CD-1 nude mice bearing U87-MG-Luc2 xenograft taken on Day 30 after tumour implantation: a) dosed with gemcitabine (33mg/ kg on Days 4, 7 and 10) in combination with GC60 – siRNA-ITCH (siRNA, GC60 ratio = 1: 100 g/g) and dosed with 0.081 mg/ kg siRNA daily on Days 4 to 10 inclusive, b) dosed with gemcitabine (33mg/ kg on Days 4, 7 and 10) plus GC60 - scrambled siRNA (prepared and dosed in exactly the same way as the siRNA-ITCH formulations), c) dosed with GC60 – siRNA-ITCH (siRNA, GC60 ratio = 1: 100 g/g) and dosed with 0.081 mg/ kg siRNA daily on Days 4 to 10 inclusive, d) dosed with normal saline (0.03 mL daily). Scale bar = 2 mm.

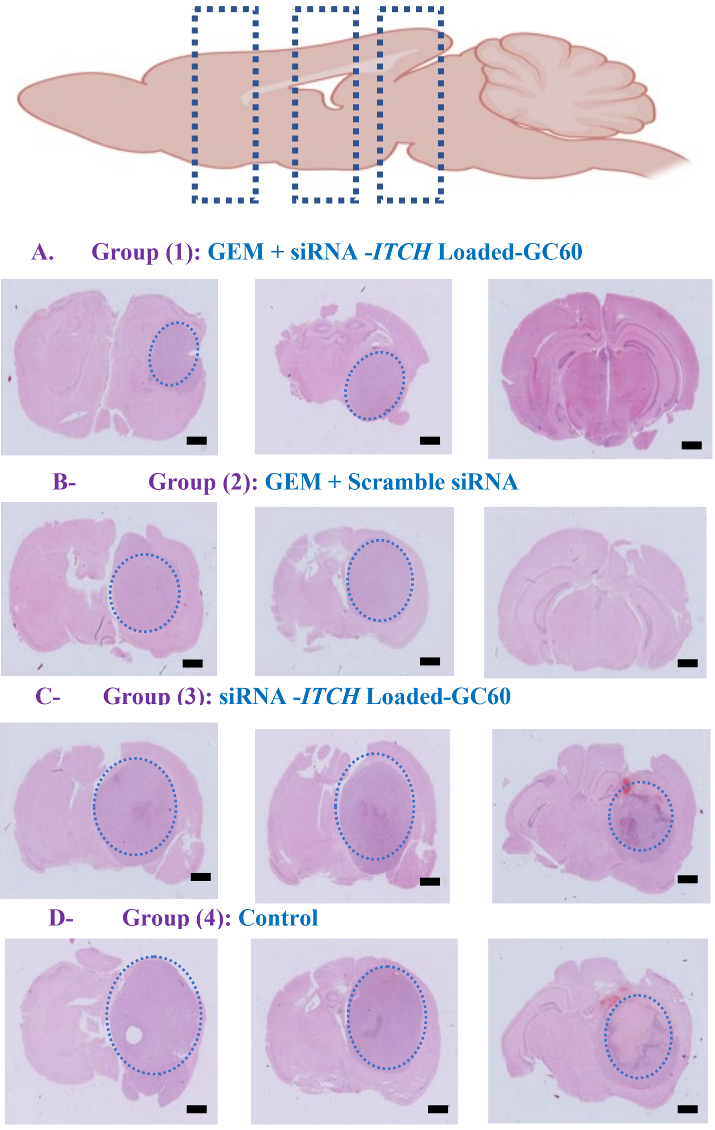

Figure S5: Representative IVIS luminescence intensity images taken at Day 25 post-tumour implantation for 4 groups of animals (n = 4) intranasally dosed with various formulations: Group 1 dosed with normal saline (0.030 mL daily), Group 2 dosed with GC60 – siRNA-ITCH (siRNA, GC60 ratio = 1: 100 g/g) and dosed with 0.081 mg/ kg siRNA daily on Days 4 to 10 inclusive, Group 3 dosed with gemcitabine (33mg/ kg on Days 4, 7 and 10) plus GC60 - scrambled siRNA (prepared and dosed in exactly the same way as the siRNA-ITCH formulations), Group 4 dosed with gemcitabine (33mg/ kg on Days 4, 7 and 10) in combination with GC60 – siRNA-ITCH (siRNA, GC60 ratio = 1: 100 g/g) and dosed with 0.081 mg/ kg siRNA daily on Days 4 to 10 inclusive.

A- Group (1): Control Normal Saline

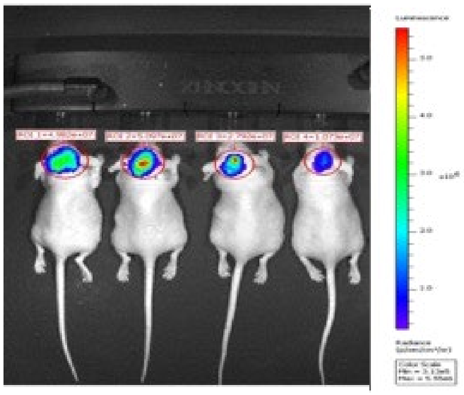

B- Group (2): siRNA -*ITCH* Loaded-GC60

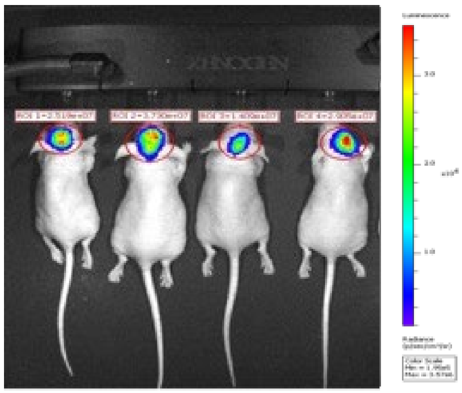

C- Group (3): GEM + Scramble siRNA

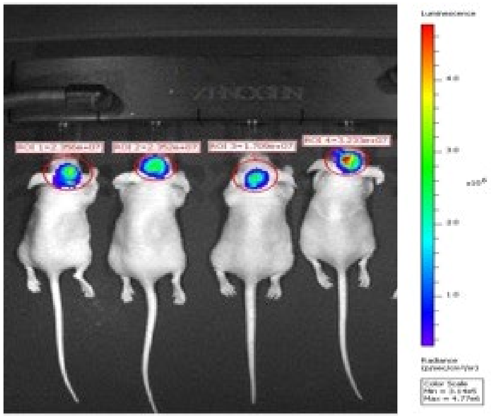

D- Group (4): GEM + siRNA -*ITCH* Loaded-

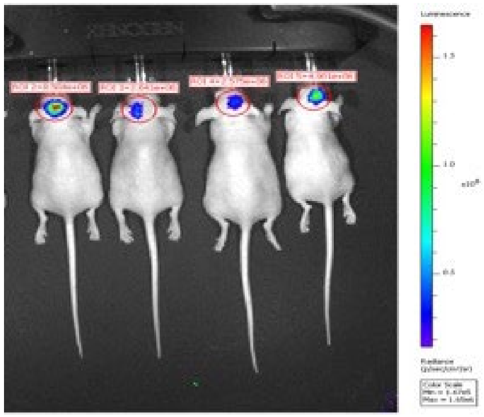

Figure S6: In vivo brain gene silencing showing down regulation of the ITCH protein from Western Blot data, as described in the Methods above.

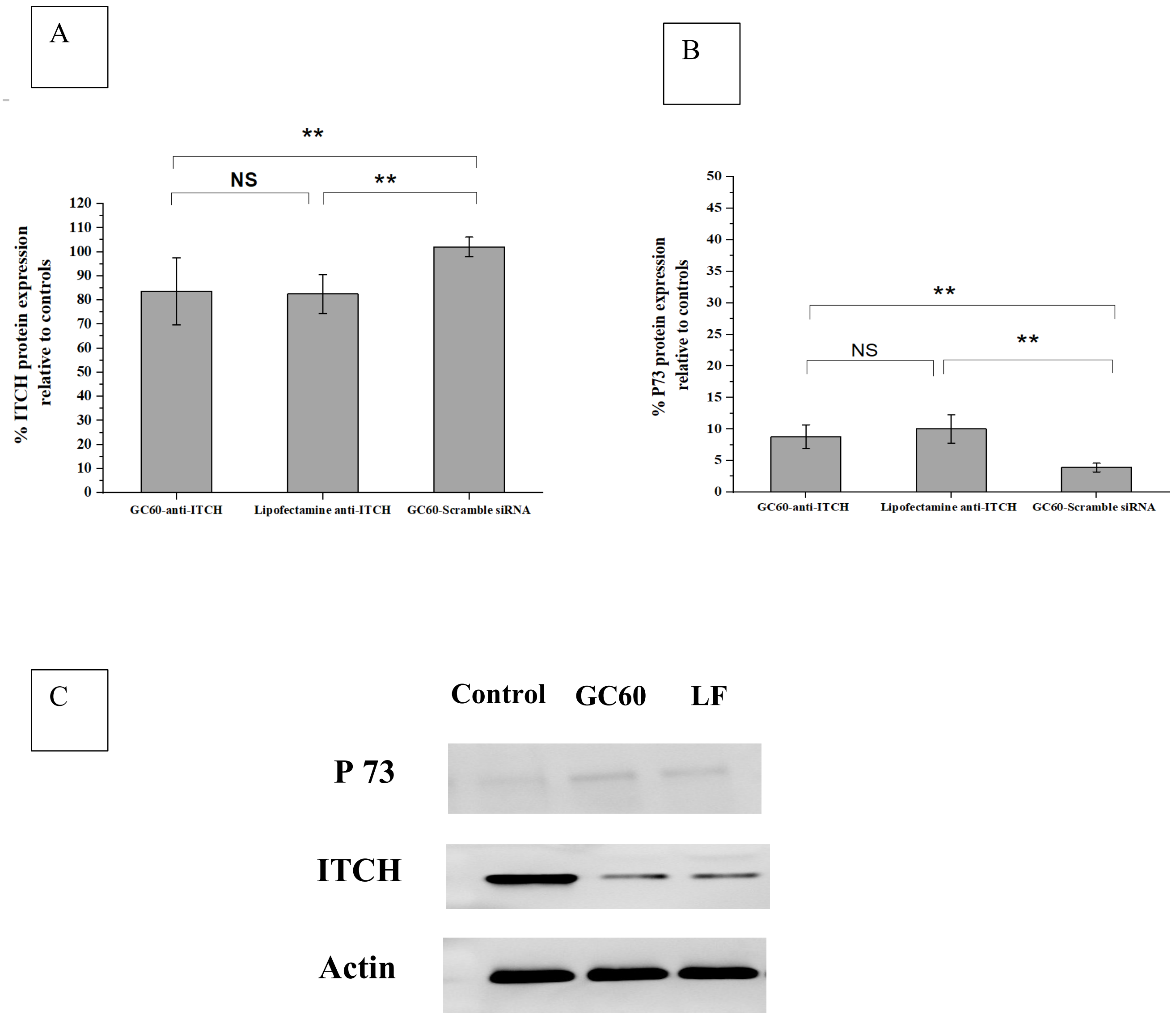

D

In vivo blot of P73

In vivo blot of Actin

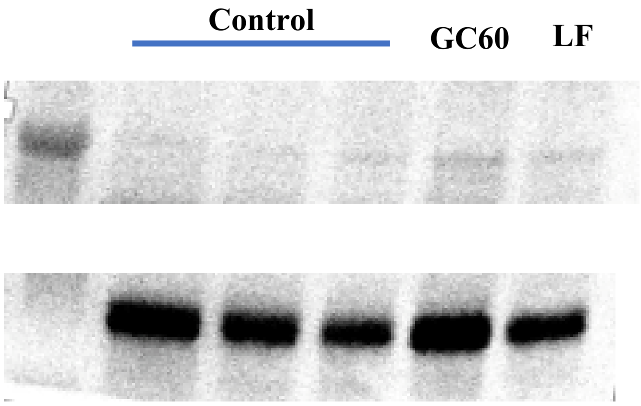

E

In vivo blot of ITCH

In vivo blot of P73

In vivo blot of Actin

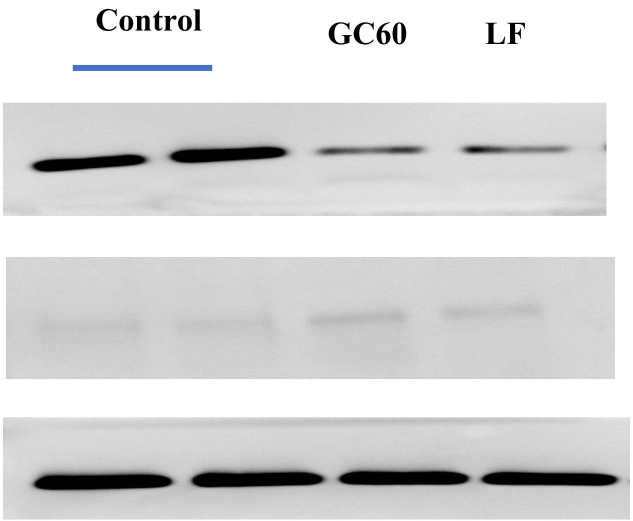

Figure S7: Raw Western Blot data showing the *in vitro* gene silencing in the U87-MG cell line. Down regulation of ITCH and the upregulation of p73 is seen and the methods are described above.

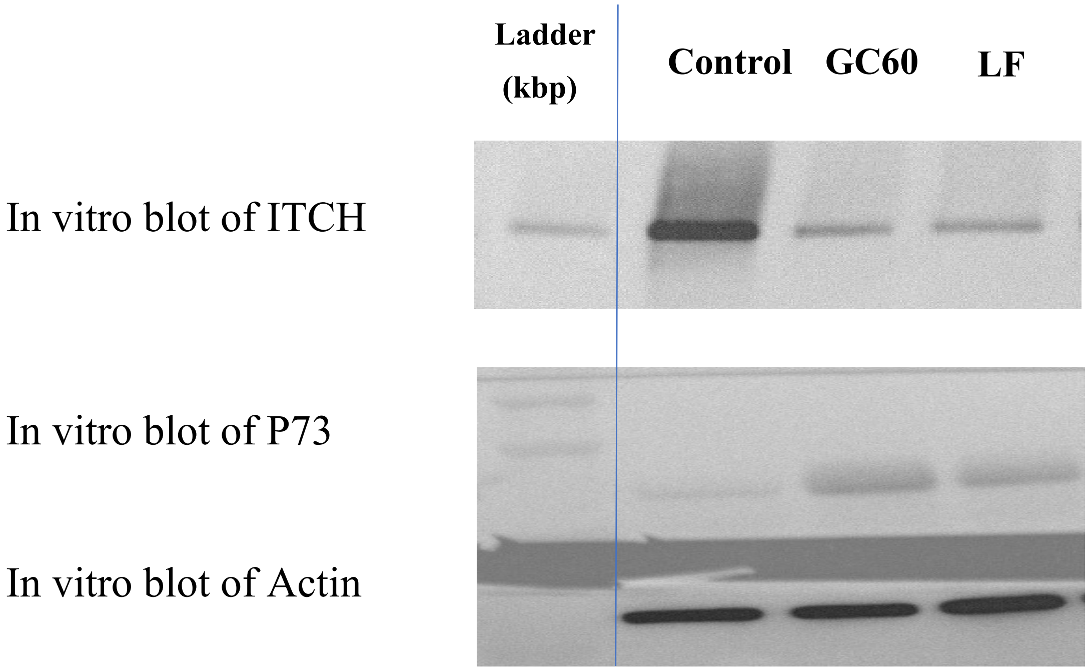

Figure S8: Absence of gross histological olfactory bulb and parenchymal tissue toxicity in CD-1 nude mice bearing the U87 MG-Luc2 group treated via the nose to brain route with the combination of GC60-siRNA-ITCH (6 doses of 0.081 mg/kg siRNA-ITCH) and gemcitabine (3 doses of 33mg/ kg). Scale bar = 2 mm for whole tissue and 100 µm for brain parenchyma images.

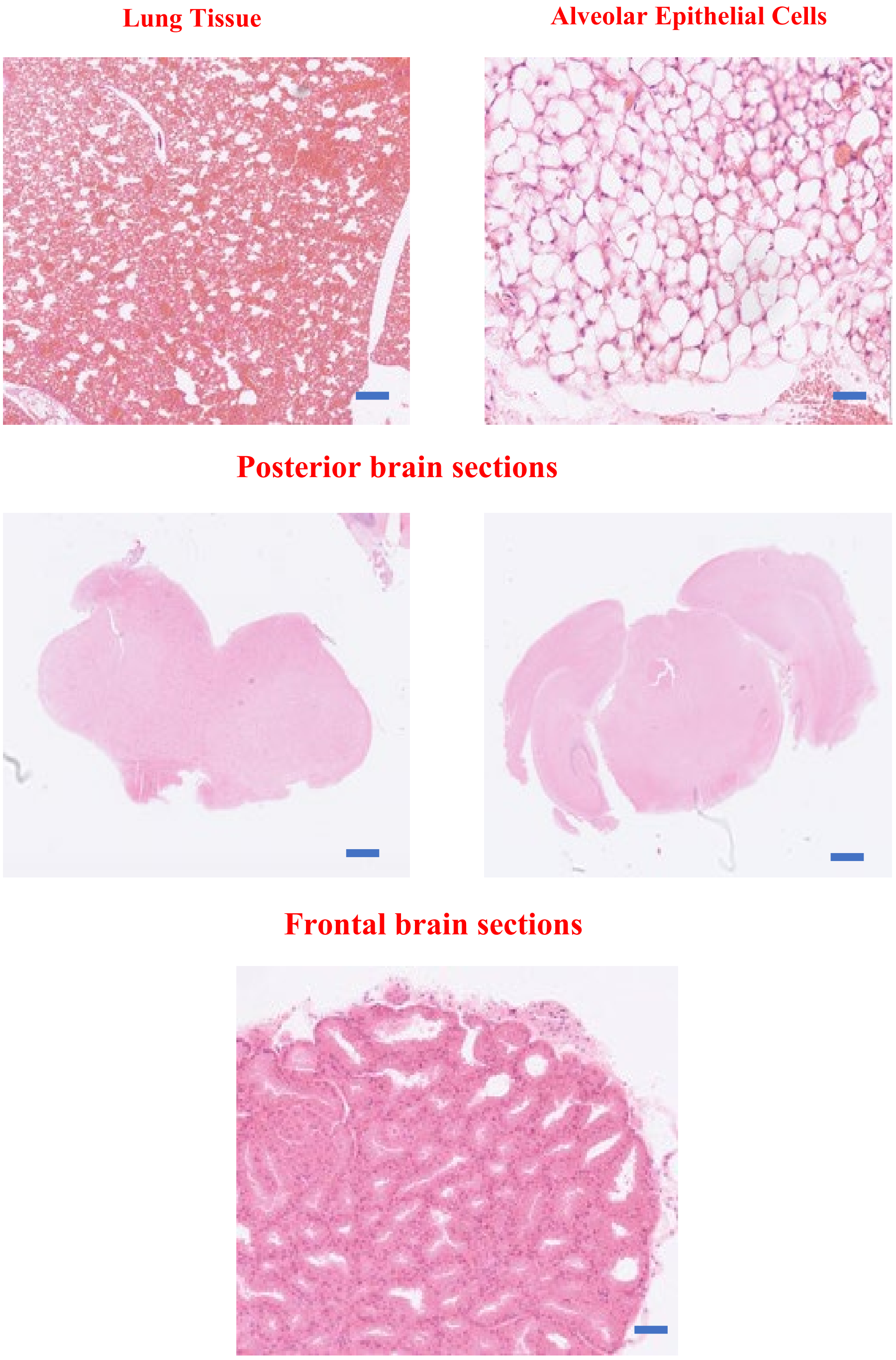

Supplement: Supplementary file 1 [file pharmaceutics-16-00829-s001.zip › pharmaceutics-3026909-supplementary.pdf]
